# A continent-wide high genetic load in African buffalo revealed by clines in the frequency of deleterious alleles, genetic hitchhiking and linkage disequilibrium

**DOI:** 10.1371/journal.pone.0259685

**Published:** 2021-12-09

**Authors:** Pim van Hooft, Wayne M. Getz, Barend J. Greyling, Bas Zwaan, Armanda D. S. Bastos

**Affiliations:** 1 Wildlife Ecology and Conservation Group, Wageningen University, Wageningen, The Netherlands; 2 Department of Environmental Science Policy & Management, University of California, Berkeley, California, United States of America; 3 School of Mathematical Sciences, University of KwaZulu-Natal, Durban, South Africa; 4 Agricultural Research Council, Irene, South Africa; 5 Laboratory of Genetics, Wageningen University, Wageningen, The Netherlands; 6 Department of Zoology & Entomology, University of Pretoria, Hatfield, South Africa; Amity University, INDIA

## Abstract

A high genetic load can negatively affect population viability and increase susceptibility to diseases and other environmental stressors. Prior microsatellite studies of two African buffalo (*Syncerus caffer*) populations in South Africa indicated substantial genome-wide genetic load due to high-frequency occurrence of deleterious alleles. The occurrence of these alleles, which negatively affect male body condition and bovine tuberculosis resistance, throughout most of the buffalo’s range were evaluated in this study. Using available microsatellite data (2–17 microsatellite loci) for 1676 animals from 34 localities (from 25°S to 5°N), we uncovered continent-wide frequency clines of microsatellite alleles associated with the aforementioned male traits. Frequencies decreased over a south-to-north latitude range (average per-locus Pearson *r* = -0.22). The frequency clines coincided with a multilocus-heterozygosity cline (adjusted *R*^2^ = 0.84), showing up to a 16% decrease in southern Africa compared to East Africa. Furthermore, continent-wide linkage disequilibrium (LD) at five linked locus pairs was detected, characterized by a high fraction of positive interlocus associations (0.66, 95% CI: 0.53, 0.77) between male-deleterious-trait-associated alleles. Our findings suggest continent-wide and genome-wide selection of male-deleterious alleles driven by an earlier observed sex-chromosomal meiotic drive system, resulting in frequency clines, reduced heterozygosity due to hitchhiking effects and extensive LD due to male-deleterious alleles co-occurring in haplotypes. The selection pressures involved must be high to prevent destruction of allele-frequency clines and haplotypes by LD decay. Since most buffalo populations are stable, these results indicate that natural mammal populations, depending on their genetic background, can withstand a high genetic load.

## Introduction

The presence of deleterious alleles in wildlife populations can result in genetic load: a decrease of the fitness of the average genotype relative to the fittest genotype present in a population [1]. A high genetic load can strongly increase local extinction risk via inbreeding depression, particularly in small populations [2, 3]. Inbreeding depression, which is characterized by lower fertility, survival and growth rates, is generally caused by increased homozygosity for partially recessive deleterious mutations (dominance hypothesis) [4]. However, a high genetic load does not necessarily results in a reduced population growth rate [3, 5]. It has been argued that in many cases deleterious alleles have a limited impact because of density-dependent processes [6]. According to this argumentation selection is typically soft, where selective death due to deleterious alleles would otherwise be replaced by nonselective death due to environmental and ecological conditions (e.g., droughts and intraspecific resource competition). Consequently, population size and persistence often do not seem to be affected by the presence of deleterious alleles, even when they are clearly expressed [7].

Even in the absence of clear effects on population viability, increased expression of deleterious alleles via inbreeding can reduce immune function and body condition in wildlife, thereby increasing susceptibility to pathogens [8–10]. Examples are gastrointestinal nematodes in Soay sheep (*Ovis aries*), hookworm, helminth and bacterial infections in California sea lions (*Zalophus californianus*), feather lice parasitism in wild lesser kestrel (*Falco naumanni*) and Galapagos hawk (*Buteo galapagoensis*), avian malaria in crows (*Corvus brachyrhynchos*) and blue tits (*Cyanistes caeruleus*), West Nile virus in crows (*Corvus brachyrhynchos*), bovine tuberculosis (BTB) in badgers (*Meles meles*), red deer (*Cervus elaphus*) and lions (*Panthera leo*), and porcine circovirus type 2 in wild boar (*Sus scrofa*) [8, 10–19].

African buffalo (*Syncerus caffer*) is a suitable species for studying wildlife population viability in the presence of high genetic load. Most buffalo populations in southern Africa are seemingly thriving despite the observation of high frequencies of expressed deleterious alleles in Kruger National Park (KNP) and Hluhluwe-iMfolozi Park (HiP), South Africa [20–22]. High frequencies of deleterious alleles in these two populations, however, are a result of a sex-ratio meiotic gene-drive system rather than inbreeding [20, 21]. Meiotic drivers are selfish genetic elements that, by distorting meiosis, favour transmission of the chromosome on which they reside. In the case of sex chromosomes, this results in distorted primary sex ratios [23].

Several microsatellite studies on African buffalo from KNP and HiP presented multiple independent lines of evidence, summarized in S1 Table, for the occurrence of deleterious alleles with a negative effect in males on body condition and BTB resistance [20–22, 24, 25]. Two types of microsatellites were observed: one type containing alleles associated with negative phenotypic effects in both sexes, and another containing alleles associated with negative phenotypic effects in males but positive phenotypic effects in females. As these microsatellite alleles are likely linked to male-deleterious alleles at protein-coding genes we will henceforth refer to these as male-deleterious-trait-associated (MDTA) microsatellite alleles. Considering the notable frequency differences among year-cohorts and between unhealthy (BTB-positive and low body condition) and healthy (BTB-negative and high body condition) males, these MDTA alleles likely occur genome-wide and at relatively high frequencies in both the KNP and HiP population.

**Fig 1:**
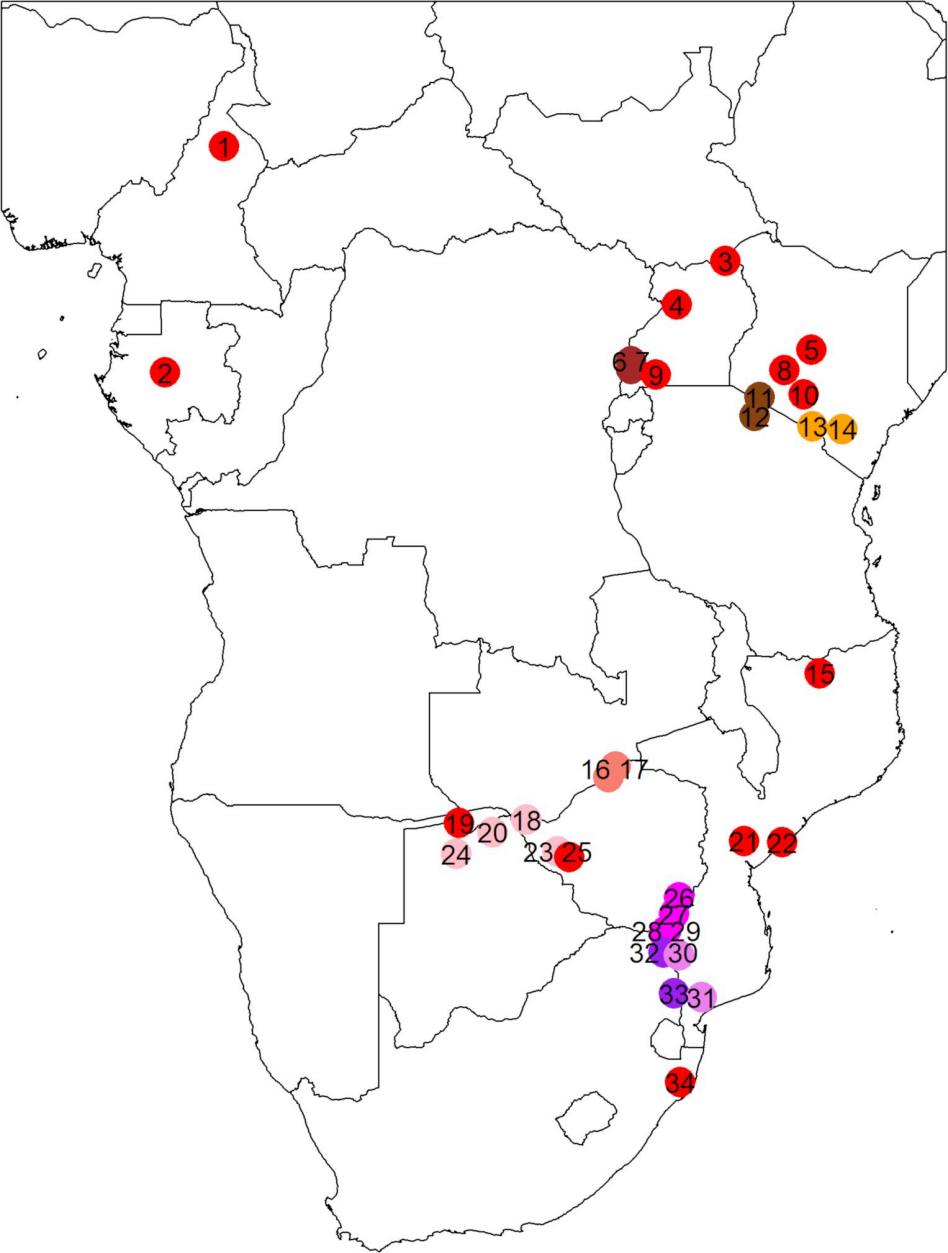
Map of Africa representing the 34 sampling localities.

Male-deleterious alleles appear to have high frequencies and be under positive selection in KNP despite the negative phenotypic effects [22]. Positive selection has been attributed to a sex-ratio meiotic gene-drive system that appears also to be health-associated (S1 Table) [20–22, 25]. It has been hypothesized that poor health in males (due to low body condition and BTB infection) suppresses sex-ratio distortion genes in this gene-drive system that, when active, reduces fertility. As a consequence, any allele that has a negative effect on male health may have a positive effect on the relative fertility of males, thereby being under positive selection if the net fitness effect of health and fertility across both sexes is positive. In contrast to positive selection of male-deleterious alleles in KNP, however, selection of male-deleterious alleles appears to be negative in HiP (situated just 280 km further south), resulting in relatively low male-deleterious allele frequencies compared to KNP [20]. As discussed elsewhere, this negative selection has been attributed to incompleteness of the gene-drive system [20, 21]. The hypothesized sex-ratio meiotic gene-drive system and its relationship with male-deleterious alleles have been clarified in Van Hooft et al. 2019 (Table 1 and Fig 1 and S1 Text herein) [20].

**Table 1:** Overview of African buffalo populations included in this study.

Population no.	Population	Lat.	Lon.	μsat set	Sample size
1	Bénoué NP	8.33	13.83	B	3
2	Lopé NP	-0.50	11.50	B	2
3	Kidepo Valley NP	3.85	33.75	C	19
4	Murchison Falls NP	2.15	31.81	C	15
5	Laikipia NP	0.40	37.16	C	17
6	Queen Elizabeth NP Mw^a^	-0.06	30.00	C	37
7	Queen Elizabeth NP Is^a^	-0.36	30.00	C	17
8	Lake Nakuru NP	-0.40	36.10	B, C	35, 18
9	Lake Mburo NP	-0.58	30.99	C	15
10	Nairobi NP	-1.36	36.85	C	19
11	Masai Mara GR	-1.47	35.10	B, C	10, 33
12	Serengeti NP^b^	-2.20	34.90	B, E	35, 49
13	Amboseli NP	-2.60	37.20	B, C	20, 19
14	Tsavo NP	-2.70	38.40	B	9
15	Niassa Reserve	-12.25	37.50	D	20
16	Mana Pools NP	-15.90	29.40	D	10
17	Nyakasanga	-16.30	29.10	D	2
18	Victoria Falls NP	-17.97	25.85	D	15
19	Caprivi Strip	-18.10	23.17	F	134
20	Chobe NP	-18.45	24.50	D	22
21	Gorongosa NP	-18.80	34.50	D	7
22	Marromeu GR	-18.85	36.00	D	21
23	Hwange NP	-19.20	27.10	D	6
24	Okavango Delta	-19.30	23.05	D	20
25	Save Valley Conservancy^c^	-19.43	27.54	B	10
26	Malilangwe Wildlife Res.	-21.00	31.90	D	20
27	Gonarezhou NP	-21.65	31.70	D	42
28	Sengwe Safari Area	-22.30	31.40	D	8
29	Crooks corner	-22.42	31.31	D	13
30	Limpopo NP	-23.25	31.90	D	6
31	Manguana	-24.90	32.80	D	4
32	Northern KNP^d^	-23.15	31.30	A, B, D	138, 22, 26
33	Southern KNP^d^	-24.75	31.70	A, B	321, 16
34	HiP^e^	-28.22	31.95	A, D	401, 20

a: Mweya sector and Ishasha sector,

b: Including Maswa GR,

c: Intermediate coordinates, which refer to the origin of the buffalos, weighted by stock size. Buffalo were restocked in 1993 from Gonarezhou NP (38 animals) and Hwange NP (360 animals) [33],

d: Kruger National Park, north and south of Olifants River,

e: Hluhluwe-iMfolozi Park

It is unlikely that the male-deleterious alleles are restricted to just KNP and HiP. Positive selection and movement of individuals (both diffusive and migratory) may have spread these alleles, together with the linked (hitchhiking) MDTA alleles, across a large geographic range. The interplay between gene flow and geographic variation in selection pressure may have resulted in allele-frequency clines [26–28]. Such clines have previously been observed in KNP, not only for the MDTA alleles but also for a Y-chromosomal haplotype hypothesized to be linked to a suppressor gene from the gene-drive system [22].

Multilocus selection may have had a negative effect on population-level genetic diversity by hitchhiking of loci linked to the male-deleterious alleles in a process known as selective sweep [29, 30]. Multilocus selection may also have resulted in extended linkage disequilibrium (LD) due to increased frequencies of haplotypes containing multiple male-deleterious alleles relative to frequencies of haplotypes expected under linkage equilibrium [31]. This may be particularly so if sex-specific selection, a potential consequence of the aforementioned sex-ratio meiotic gene-drive system, resulted in admixture LD due to different allele frequencies in male and female gametes [32]. The latter is supported by the observation of significant genetic differentiation between male and female calves in KNP [21].

Confirmation of an expansive distribution of expressed male-deleterious alleles is of concern as it would suggest that many African buffalo populations experience a high genetic load, making them sensitive to infectious diseases and other environmental stressors. In this study, we address this concern through analysis of previously published microsatellite data from 1676 buffalo from 34 localities across the African continent (Fig 1). Implicit in our analyses is the assumption that microsatellite alleles occurring at a higher frequency among BTB-positive males of low body condition than among BTB-negative males of high body condition tend to be associated with a male-deleterious allele (i.e., no significance to be expected if this assumption is wrong). With this assumption, we addressed the following three questions:

1. Did selection result in male-deleterious allele-frequency clines across the geographic area under study?
2. Did selection, via genetic hitchhiking, result in decreased population-level genetic diversity?
3. Did selection result in increased LD between male-deleterious alleles?

## Materials and methods

### Localities and microsatellites

We analysed 1676 animals from 34 localities throughout the buffalo’s range (Fig 1 and Table 1). These localities span approximately 37 degrees of latitude (± 4500 km from Bénoué NP in Cameroon to HiP in South Africa) and 27 degrees of longitude (± 3000 km from Lopé NP in Gabon to Tsavo NP in Kenya).

We used previously published genotype data of the following seventeen microsatellites, originally developed for cattle (*Bos taurus*), which were randomly chosen with respect to genomic location: *BM0719*, *BM1824*, *BM3517*, *BM3205*, *BM4028*, *CSSM019*, *DIK020*, *ETH010*, *ETH225*, *ILSTS026*, *INRA006*, *INRA128*, *SPS115*, *TGLA057*, *TGLA0159*, *TGLA227* and *TGLA263* [20–22, 33–38]. Data for all seventeen microsatellites were only available for KNP and HiP (microsatellite set A in Table 2). Five smaller subsets, consisting of 2–11 microsatellites, were available for the other populations (microsatellite sets B-F in Table 2). Raw microsatellite data from microsatellite sets D and F are provided in earlier publications [34, 37], while those from the remaining sets are available from the Dryad Digital Repository [39].

**Table 2:** Overview of microsatellite sets used.

Set	μsats	Reference
A	1–17	[20, 22, 38]
B	1, 2, 5, 6, 8–12, 14–16	[33]
C	1, 2, 6, 9–12, 14–16	[35]
D	2–10, 13, 15, 17	[34]
E	3, 13, 17	[36]
F	1, 11	[37]

1: *BM3517*, 2: *BM4028*, 3: *ETH010*, 4: *ETH225*, 5: *INRA006*, 6: *INRA128*, 7: *TGLA227*, 8: *TGLA263*, 9: *CSSM019*, 10: *DIK020*, 11: *TGLA057*, 12: *BM0719*, 13: BM1824, 14: *BM3205*, 15: *ILSTS026*, 16: *TGLA159*, 17: *SPS115*.

### Allele size standardization

Genotype data from different studies obtained with the aforementioned six microsatellite sets were combined after standardizing allele sizes using the Genalex add-in for Excel (version 6.503) [40, 41]. Standardization was necessary, because allele size estimates have been observed to differ by up to 17 bp among platforms [42, 43]. In our case they differed by up to 11 bp (microsatellite set B relative to set A: *BM3517*: 8 bp, *INRA006*: 11 bp, in both cases unambiguous standardization despite large size difference; ≤ 6 bp in the remaining cases). For various populations, two microsatellite sets were available (sets A-D in Tables 2 and 3; sample size per set per population: ≥ 38 individuals), which permitted allele alignment by matching (allele size shift) each microsatellite’s allele frequencies while preserving size order. Aligning microsatellite alleles based on frequency information is a valid standardization method when data sets are reasonably large and coming from the same population [44–48]. With two microsatellites sets (sets E and F in Tables 2 and 3; sample size per set per population: ≥ 38 individuals) standardization was only possible in comparison with another microsatellite set applied to another population. Because of the higher risk of alignment errors in the latter case, we also conducted the tests for allele-frequency clines and for correlation between heterozygosity clines and allele-frequency clines without these two microsatellite sets (see Results section).

**Table 3:** Linkage disequilibrium in Kruger National Park among locus pairs showing significant LD in earlier studies.

Locus pair	multi-allelic |*r*_LD_|^a^	p-MDTA *r*_LD_	p-random *r*_LD_^b^	Exact probability^c^
*BM1824*-*BM3205*	0.126 (0.358)	0.176	0.031	0.28
*BM1824*-*CSMM019*	0.157 (0.189)	0.337	0.076	0.17
*CSMM019*-*BM3205*	0.158 (0.154)	0.339	0.031	0.033
Average first three pairs	0.147 (0.234)	0.284	0.046	0.010
*ILSTS026*-*INRA006*	0.128 (0.141)	-0.041	0.002	0.76
Average all pairs	0.142 (0.211)	0.203	0.035	0.019

a: Between brackets: multi-allelic |*r*_LD_| in Hluhluwe-iMfolozi Park,

b: average *r*_LD_ when alleles were randomly assigned to the focal allele class,

c: exact 2 times 1-sided probability p-random ≥ p-MDTA (*ILSTS026*-*INRA006*: p-random ≤ p-MDTA)

The allele size shift we used was the one that resulted in the highest Pearson correlation in allele frequencies and lowest *F*_ST_ between two sets. Given large enough sample sizes, their values are expected to be close to 1 and 0, respectively. Generally, the obtained Pearson correlations were > 0.8 and the *F*_ST_ values < 0.01. A detailed description of the allele size standardization procedure is given in S1 Text and S2 Table.

Unaccounted for alignment errors probably increased type II errors and were unlikely to have resulted in a systematic bias of allele frequency estimates. Accuracy of the allele size standardization was strongly supported by the observation that frequency clines of p-MDTA alleles correlated with heterozygosity clines, of which the latter were not influenced by possible alignment errors (see Results section). Furthermore, it is unlikely that alignment errors generally resulted in positive LD among p-MDTA alleles, as was also observed in KNP with microsatellite set A only (see Results section). Additional support for the accuracy of the allele size standardization was provided by the high amount of explained variation among populations in East and southern Africa, with *R*^2^ being 0.78, when regressing pairwise multilocus *F*_ST_ with geographic distance (predicted *F*_ST_ increasing from 0.014 at 0 km to 0.11 at 3500 km, S1 Fig).

### Male-deleterious-trait associations

Alleles at the seventeen microsatellite loci (dataset A) have previously been shown to be significantly associated with two deleterious traits in male buffalo from southern KNP (i.e., south of the Olifants River): 1) low body condition and 2) BTB infection (see also S1 Table) [21, 22]. BTB was present only in southern KNP at the time of sampling, except for three BTB-positive individuals in the north. Here we considered all microsatellite alleles from southern KNP, after excluding rare alleles, as being associated with male-deleterious traits when they had a higher frequency among low-body-condition, BTB-positive males than among high-body-condition, BTB-negative males (S3 Table). The sample sizes of these two male groups were similar, *N* = 35 and *N* = 39 respectively, with alleles considered rare when observed ≤ 8 times (frequency ≤ 0.054) in total in these two groups. This was the highest possible cut-off point where both male-deleterious-trait-associated (MDTA) alleles and wild-type associated alleles (relatively low frequency among low-body-condition, BTB-positive males) could be identified at each locus (32 MDTA and 36 wild-type associated alleles in total, S3 Table). As argued in an earlier study, comparison of allele frequencies between the two male groups probably constitutes a reliable indicator of association strength with male-deleterious traits [20]. In all tests, we pooled the frequencies of MDTA alleles at each microsatellite locus (S4 Table). Thus, as discussed elsewhere [20], we assumed two alleles per coding gene: a male-deleterious allele associated with the pooled MDTA (p-MDTA) alleles and a wild-type allele associated with the pooled remaining (p-wildtype) microsatellite alleles.

### Allele-frequency clines

We tested whether allele-frequency clines could be attributed to selection by comparing frequency clines of p-MDTA alleles with frequency clines of randomly pooled (p-random) microsatellite alleles. Microsatellite alleles were randomly pooled at each locus with the same number of constituent alleles per locus as the MDTA alleles (without changing the original genotypes). The frequency clines were based on the frequency differences per locus between p-MDTA and p-wildtype alleles (p-MDTA minus p-wildtype) for each of 31 localities. We excluded HiP (location 34 in Fig 1 and Table 1), because here the male-deleterious alleles had earlier been shown to be under strong negative selection [20]. Samples from Bénoué NP and Lopé NP (separated by ± 1000 km; locations 1–2) in central Africa and from Mana Pools NP and Nyakasanga (separated by ± 55 km; locations 16–17) in southern Africa were pooled because of small sample size. Intermediate coordinates, weighted by sample size, were used for these pooled samples. We estimated significance as the probability that random pooling of alleles resulted in an average Pearson correlation (*r*) between frequency difference and latitude at each locus at least strong as with the p-MDTA alleles. To minimize bias in average *r* we first transformed each individual *r* to a Fisher’s *z*, after which we back-transformed the average *z* [49]. This transformation did not affect probability estimates. Further, to reduce heteroskedasticity, the *r* values for each locus were weighted by the square-root of the number of individuals genotyped at the locus at each location. We weighted by the square-root because without this transformation the *r* values would have been strongly biased by the relatively large sample sizes of the two KNP subpopulations.

We also determined whether per-locus allele-frequency differences (p-MDTA minus p-wildtype) were significantly positive in both central-East and southern Africa. If so, this positivity would be indicative of selection across the entire latitudinal range. Again, allele-frequency differences were weighted by the square-root of the number of genotypes per location. We estimated significance as the probability that random pooling of alleles resulted in an average allele-frequency difference per locus at least as high as with the p-MDTA alleles.

In both tests, we applied 100,000 randomizations implemented using Excel 2016. The *P*-values are reported as 2 times 1-sided values. We plotted average per-locus frequency difference against latitude, with the linear regression line obtained by weighing the population at each location by the square-root of the number of genotyped individuals multiplied by the average number of genotyped loci per individual.

### Heterozygosity clines

We conducted two statistical tests for an effect of selection on expected heterozygosity (*H*_e_), with *H*_e_ estimates at each locus based on the original non-pooled microsatellite alleles. Unbiased *H*_e_ estimates [50] were obtained with the Excel add-in Microsatellite Toolkit [51]. To make *H*_e_ estimates independent from assumptions about allele size standardization we only used microsatellite set A for KNP and used the weighted average *H*_e_ of microsatellite sets B and C (i.e., *H*_e_ was separately estimated for each set) for Lake Nakuru NP, Masai Mara GR and Amboseli NP. Only one microsatellite set was available for each of the remaining localities. We would like to stress here that the *H*_e_ estimates were unaffected by any assumptions about male-deleterious alleles.

In the first heterozygosity test, we determined whether selection had an influence on population-level *H*_e_ by correlating heterozygosity clines with the aforementioned allele-frequency clines. A negative correlation would indicate a negative influence. As before, we excluded HiP (location 34 in Fig 1 and Table 1) because of earlier observed negative selection [20]. We also excluded Bénoué NP (location 1), Lopé NP (location 2) and Nyakasanga (location 17) because of small sample size and the admixed population from Save Valley Conservancy (location 25; buffalo were restocked in 1993 with 38 animals from Gonarezhou NP and 360 animals from Hwange NP [33]) because admixing may positively bias *H*_e_ estimates. We estimated significance as the probability that random pooling of alleles resulted in a Pearson correlation between the *H*_e_ cline (correlation between *H*_e_ and latitude) and the allele-frequency cline (correlation between allele frequency and latitude) at each locus at least as strong as the correlation between the *H*_e_ cline and the observed p-MDTA allele-frequency cline. We applied 100,000 randomizations implemented using Excel 2016. The *P*-values are reported as 2 times 1-sided values.

We plotted multilocus *H*_e_ against latitude, including only localities for which the same microsatellites were analysed and with the linear regression line obtained by weighing the population at each location by the square-root of the total number of genotypes. One group of seventeen localities between 24.9°S and 2.2°S and a second group of twelve localities between 2.7°S and 3.9°N were selected for analysis using ten microsatellites for each group, with five microsatellites shared between the two groups. Multilocus *H*_e_ estimates in the southern group were based on microsatellites *BM1824*, *BM4028*, *ETH010*, *INRA006*, *INRA128*, *CSSM019*, *DIK020*, *ILSTS026*, *SPS115* and *TGLA263*; and those in the eastern group on microsatellites *BM3517*, *BM4028*, *INRA128*, *BM0719*, *BM3205*, *CSSM019*, *DIK020*, *ILSTS026*, *TGLA057* and *TGLA159*. Multilocus *H*_e_ of the northern localities was multiplied with 0.966, which was the ratio between the two microsatellite sets in Serengeti NP; the only locality in East Africa analysed with both sets.

In the second heterozygosity test we determined whether recent (since < 40 years before sampling) and historical (since > 100 years before sampling) selection pressures were correlated. In particular, we conducted a Spearman correlation between the per-locus southern/northern KNP *H*_e_ ratio (*H*_e_ southern KNP/*H*_e_ northern KNP), indicative of a short-term hitchhiking effect caused by the introduction of BTB around 1960 [21, 22], and the weighted Pearson correlation (by square-root of number of genotypes) between *H*_e_ and latitude across Africa (24.9°S—3.9°N, excluding southern KNP for independence with aforementioned *H*_e_ ratio), indicative of a long-term hitchhiking effect. A significant relationship cannot be attributed to random genetic drift considering that in such case the fractional decrease in per-locus *H*_e_ would be determined by effective population size and gene flow, which are not locus specific. Despite a small genetic distance between northern and southern KNP (*F*_ST_ = 0.0034, 95% CI = 0.0023, 0.0045; estimated with FSTAT 2.9.3 [52, 53]), the latter had a significantly lower multilocus *H*_e_ (multilocus *H*_e_ northern KNP = 0.66, multilocus *H*_e_ southern KNP = 0.64; Student’s paired t-test: *P* = 0.014, *N* = 17).

### Linkage disequilibrium

Four microsatellite pairs showed highly significant LD in both KNP and HiP [20, 22]. Three of these occur close on chromosome 1 in cattle and its homologous chromosome in African buffalo (whole genome sequence data of cattle and contig sequence data of buffalo at NCBI website): *BM1824*-*CSMM019* (0.5 Mb in buffalo; microsatellite sets A and D in Table 2), *CSSM01*9-*BM3205* (5.4 Mb; sets A-C), *BM1824*-*BM3205* (5.9 Mb; set A) [54]. The fourth pair consists of microsatellites *ILSTS026* and *INRA006* (sets A, B and D), which are probably ~19 Mb apart on the same chromosome in buffalo. This chromosome corresponds to a fusing of chromosomes 2 and 3 in cattle, with the former harbouring *ILSTS026* and the latter *INRA006*, in both cases ~9.5 Mb from the end of the left-side of their p arms [54–56]. Although 19 Mb is still a relatively large distance, recombination rate may well be reduced in the fused region [57]. Two additional microsatellite pairs are at close chromosomal distance, although they did not show significant LD in KNP or HiP: *INRA006*-*TGLA263* (27.5 Mb in buffalo, chromosome 3 in cattle; microsatellite sets A, B and D in Table 2) and *TGLA057*-*INRA128* (28.3 Mb, chromosome 1 in cattle; sets A-C) [54]. The chromosomal distances at the aforementioned locus pairs (range: 0.5–28.3 Mb) correspond to recombination rates of 0.6–31%/generation, based on a recombination rate of 1.1 cM/Mb at chromosomes 1 to 3 in cattle.

We conducted three statistical tests for LD, with LD estimated per population per locus pair, assuming two alleles per locus: the p-MDTA and the p-wildtype allele. Here, the latter also included the rare original microsatellite alleles (observed ≤ 8 times among low-body-condition, BTB-positive males and high-body-condition, BTB-negative males from southern KNP) because in LD analyses frequencies must add up to 1 (in contrast to the frequency-cline tests where rare alleles were excluded).

We measured LD as the Pearson correlation coefficient, *r*_LD_, for a 2x2 table representing bi-allelic locus pairs. When the linkage phase of all genotypes is known, *r*_LD_ can be derived from the observed and expected haplotype frequencies (*χ*^2^ value, four possible haplotypes) and the total number of gametes or chromosomes analysed (*k*), using the following equation [28]:

$$r_{LD}=\sqrt{\frac{\chi^{2}}{k}}$$ (1)

A negative sign is added to *r*_LD_ when pairs of focal alleles occur less often than expected (i.e. when they tend to be in repulsion). However, when the linkage phase of double heterozygotes is unknown, such as in our case, *r*_LD_ can be derived from maximum-likelihood estimates of the haplotype frequencies (ML *r*_LD_) [58, 59]. We estimated ML *r*_LD_ using Genalex, following Weir’s method and assuming Hardy-Weinberg equilibrium [59]. In our case, positive ML *r*_LD_ values indicate that pairs of male-deleterious alleles tend to be in coupling.

Except for KNP, unbiased estimates of *r*_LD_ were not possible, because for this a minimum sample size of 50 non-double heterozygotes is required, particularly with interlocus distances > 10 Mb [60, 61]. Therefore, we determined only the sign of *r*_LD_ in the first two LD tests and estimated significance by randomizing genotypes and alleles. We excluded populations with less than five non-double heterozygotes (i.e., < 10 known haplotypes). Any bias or inaccuracy due to low sample size equally applied to the observed and the randomized data and therefore was unlikely to affect probability estimates. We pooled the samples from the neighbouring populations in Mana Pools NP-Nyakasanga (~55 km distance; locations 16–17 in Fig 1 and Table 1) and in Limpopo NP-Manguana (~200 km distance; locations 30–31) to increase sample size. Intermediate coordinates, weighted by sample size, were used for these pooled samples. Further, we excluded HiP (location 34) because of earlier observed negative selection [20] and the admixed population from Save Valley Conservancy (location 25) because admixing may bias LD estimates.

The sign of the ML *r*_LD_ was always identical to that of *r*_LD_ for the non-double heterozygotes alone, except when the latter was close to zero and one haplotype from each of the two possible double-heterozygous haplotype pairs (one in coupling and one in repulsion) was rare. Such cases, as well as cases where *r*_LD_ of the non-double heterozygotes was exactly zero, were excluded from our analyses (different signs: *CSSM01*9-*BM3205* in Kidepo Valley NP and *TGLA057*-*INRA128* in Masai Mara GR; *r*_LD_ = 0: *CSSM01*9-*BM3205* in Murchison Falls NP and *ILSTS026*-*INRA006* in Limpopo NP). Further, Weir’s method can only estimate the sign of ML *r*_LD_ when all four possible haplotypes are observed among the non-double heterozygotes. With only three observed haplotypes, therefore, sign of *r*_LD_ was only included if linkage phase of the double heterozygotes did not affect it (i.e., all double heterozygotes in repulsion resulted in the same sign as all double heterozygotes in coupling). The occurrence of only two haplotypes was always due to monomorphism at a locus, in which case LD cannot be estimated in principle.

LD estimates of the three closest linked locus pairs, *BM1824*-*BM3205*, *BM1824*-*CSMM019* and *CSMM019*-*BM3205*, for populations that are part of a larger metapopulation may not be independent of each other (indeed, sign was always identical when part of the same metapopulation; sign test: *P* = 0.0078, *N* = 8). We excluded *BM1824*-*BM3205* because this locus pair was only analysed in KNP. Further, to err on the conservative side, we used only one estimate per metapopulation for the other two locus pairs: the fraction of populations with positive *r*_LD_. With respect to *BM1824*-*CSMM019* and *CSMM019*-*BM3205*, the following six population location lists were considered to each constitute a separate metapopulation because of their proximity and landscape-corridor connectedness (Fig 1). Metapopulation 1: northern KNP, southern KNP (locations 32–33 in Fig 1 and Table 1); Metapopulation 2: Malilangwe Wildlife Res., Gonarezhou NP, Sengwe Safari Area, Crooks Corner (locations 26–29); Metapopulation 3: Victoria Falls NP, Chobe NP, Hwange NP, Okavango Delta (locations 18, 20, 23–24); Metapopulation 4: Amboseli NP, Tsavo NP (locations 13–14); Metapopulation 5: Masai Mara GR, Serengeti NP (locations 11–12), Metapopulation 6: Queen Elizabeth NP Mw, Queen Elizabeth NP Is (locations 6–7). We assumed independence of *r*_LD_ estimates for the other three physically linked locus pairs, even when part of the same metapopulation, because of their relatively large interlocus distances. For these locus pairs, the expected recombination rate (~21–31% per generation) likely exceeds the migration rate between nearby populations. LD estimates of the neighbouring locus pairs *BM1824*-*CSMM019* and *CSMM019*-*BM3205* in KNP, the only population in our analyses that included both these pairs, were considered independent from each other in the absence of selection, which was supported by the results of the third LD test (no meaningful correlation in *r*_LD_ between the two locus pairs when using random allele assignments).

In the first two LD tests, we hypothesized that, in comparison with random allele associations between locus pairs, pairs of p-MDTA alleles were coupled more often than expected and that these coupled pairs occurred on average more southerly than uncoupled pairs. The latter observation suggests an LD cline, although the statistical power to detect this was quite low. Even when the fraction of coupled p-MDTA allele pairs at linked microsatellites in the buffalo genome linearly decreases from a value as high as 0.8 at 24.9°S to 0.5 (expected fraction in the absence of selection) at 4.8°N, the probability of observing a significant (*α* = 0.05) or near-significant (*α* = 0.1) difference in average latitude with a Student’s *t*-test between coupled and repulsed allele pairs was only 34% and 46%, respectively (60 *r*_LD_ estimates from 24 locations using 50 randomizations).

In both LD tests significance was estimated using two randomization schemes. In the first scheme, the genotypes at one locus per locus pair were randomized for each of the 27 locations (non-randomized data: 60 *r*_LD_ estimates in total from five locus pairs and 24 locations). In the second scheme, the non-rare microsatellite alleles (observed ≥ 9 times among low-body-condition, BTB-positive males, as well as high-body-condition, BTB-negative males from southern KNP) were randomly assigned to the focal-allele class (i.e., alleles coded as 1 vs. non-assigned alleles coded as 0; without changing the original genotypes), using the same number of constituent alleles per focal allele as the MDTA alleles. While the first randomization scheme merely tested whether LD was significant or occurred significantly more often at southern localities, the second scheme tested whether this could be attributed to selection. We estimated significance as the probability that randomized data resulted in: for LD test 1, a total fraction of locus pairs (across five locus pairs and 27 locations) with positive *r*_LD_ at least as large as the observed data with the p-MDTA alleles as focal alleles; or, LD test 2, an average latitude of locus pairs with positive *r*_LD_ at least as low as the observed data with the p-MDTA alleles as focal alleles. All populations were included in the randomizations, including those whose *r*_LD_ sign could not be determined, as a result of which the subset of datapoints differed between randomizations (i.e., < 27 locations in most randomizations). We applied 100,000 randomizations implemented using Excel 2016. *P*-values are reported as 2 times the 1-sided value.

As a control, we estimated the total fraction of locus pairs with positive *r*_LD_ across all populations for: 1) the five closely linked locus pairs (excluding *BM1824*-*BM320*, which was only analysed in KNP) with the genotypes at one locus randomized per location, and 2) locus pairs with interlocus distance > 45 Mb (seven locus pairs) or that occur on different chromosomes (123 locus pairs). Both fractions are expected to be close to 0.5 in the absence of selection (with free recombination) [62]. In the first control we conducted 100,000 randomizations of the total dataset, resulting in 2,422,396 randomized *r*_LD_ values.

In the third LD test, we determined whether earlier observed LD in KNP (treated as a single population) at locus pairs *BM1824*-*BM3205*, *BM1824*-*CSMM019*, *CSMM019*-*BM3205* and *ILSTS026*-*INRA006* based on the original non-pooled microsatellite alleles [20, 22], could be attributed not only to physical proximity but also to selection. Similar to the first two LD tests, we randomly assigned microsatellite alleles to the focal-allele class according to the second randomization scheme, and we estimated significance as twice the exact probability that random assignments resulted in a *r*_LD_ value at least as high as with the p-MDTA alleles as focal alleles. We would like to stress here that this LD test was not meaningfully influenced by possible alignment errors (similar results when only microsatellite set A was included, which was used for 88% of the KNP samples). Additionally, we tested whether *r*_LD_ values based on random allele assignments were correlated between nearby locus pairs *BM1824*-*BM3205*, *BM1824*-*CSMM019* and *CSMM019*-*BM3205*. Weak correlations would indicate that in the absence of selection *r*_LD_ values at these locus pairs can be treated as (nearly) independent observations.

For comparison, we estimated multi-allelic |*r*_LD_| (sign not relevant with > 2 alleles) in KNP and HiP based on the non-pooled microsatellite alleles at the four locus pairs with earlier observed LD, according to Equation 3 in Zhao et al. 2005 and excluding alleles with frequency < 0.075 [63]. For each allele pair we estimated ML |*r*_LD_| with Genalex, following Weir’s method and assuming Hardy-Weinberg equilibrium [59]. Comparison of *r*_LD_ values based on p-MDTA alleles between KNP and HiP was not possible because of low p-MDTA frequencies in HiP, resulting in inaccurate *r*_LD_ estimates (p-MDTA frequencies: *BM1824*: 0.0059, *BM3205*: 0.065, *INRA006*: 0.028).

## Results

### Allele-frequency clines

**Fig 2:**
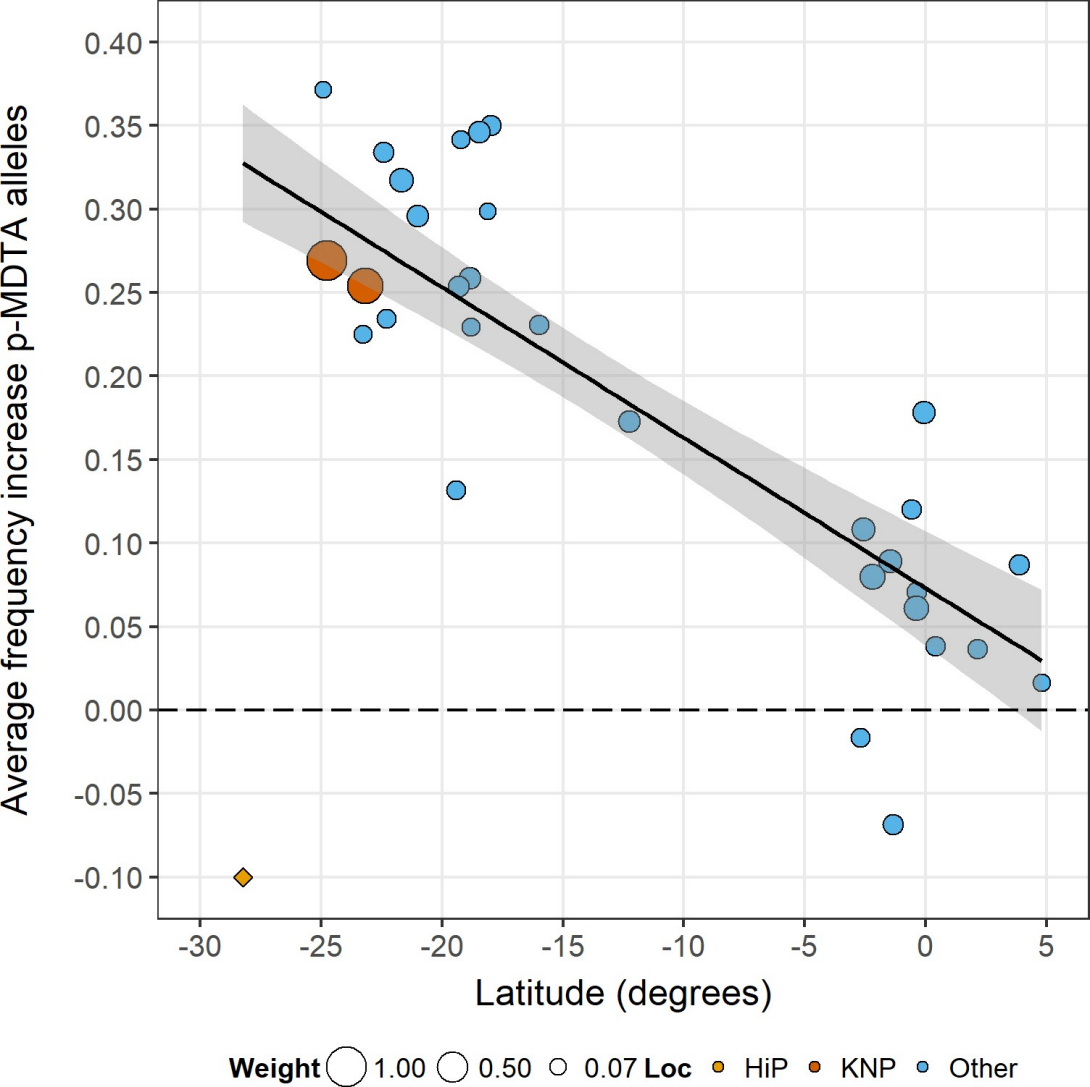
Multilocus-allele-frequency cline.

We observed allele-frequency clines between 24.9°S (treated as negative latitude values—see Fig 2) and 4.8°N with average per-locus Pearson *r* = -0.22 (frequency difference between p-MDTA and p-wildtype alleles; p-MDTA alleles: *r* = -0.53, p-wildtype alleles: *r* = -0.27), which could be attributed to selection (*P* = 0.025, 12 out of 17 loci showed a negative correlation; when excluding microsatellite sets E and F: *r* = -0.18, *P* = 0.042; S5 Table). Plotting average frequency increase of p-MDTA alleles across loci relative to p-wildtype alleles explained 74% of the variation (Fig 2). Predicted average frequency increase varied between 0.30 (95% CI: 0.27, 0.33) at 24.9°S to 0.03 (95% CI: -0.01, 0.07) at 4.8°N (a decrease of 0.0090 per degree latitude) and could be attributed to selection across the whole latitudinal range (average frequency increase; southern Africa: 0.24, *P* = 0.00095; central-East Africa: 0.08, *P* = 0.083; when excluding microsatellite sets E and F: southern Africa: 0.24, *P* = 0.00066; central-East Africa: 0.07, *P* = 0.14). The HiP population deviated from the overall trend, showing a clear frequency decrease of p-MDTA alleles relative to p-wildtype alleles (0.10 decrease) in sharp contrast to the other southern African populations (range: 0.13–0.37 increase). This frequency decrease has earlier been attributed to negative selection [20].

### Heterozygosity clines

**Fig 3:**
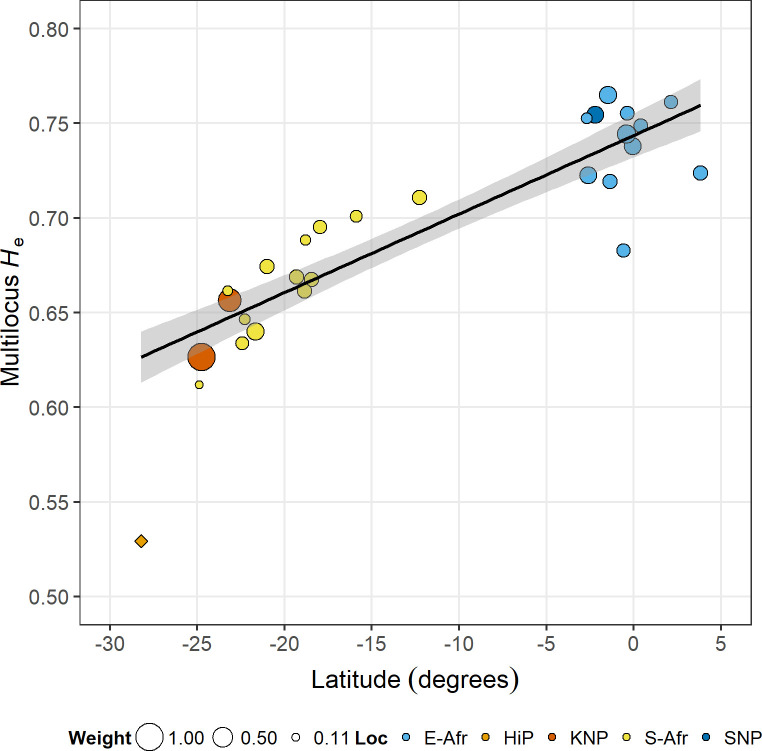
Multilocus-*H*_e_ cline.

There was a significant negative correlation between *H*_e_ clines and allele-frequency clines, which could be attributed to selection (Pearson *r* = -0.62, *P* = 0.0079, 17 loci; attribution to selection: *P* = 0.029; when excluding microsatellite sets E and F: *r* = -0.55, attribution to selection: *P* = 0.070; S5 Table). Regressing multilocus *H*_e_ against latitude explained 84% of the variation (Fig 3 and S6 Table; 85% when also including *ABS010* and *AGLA293* which were not part of microsatellite set A, but which were included in the original publications of sets B, C and D, S2 Fig; 74% when including only the seven microsatellites analysed in both East and southern Africa with sets A, B and D, plus *ABS010* and *AGLA293*, S3 Fig). Predicted multilocus *H*_e_ decreased by 16% (95% CI: 13%, 19%) at 24.9°S compared to 3.9°N. Again, the HiP population deviated from the overall trend, showing by far the lowest multilocus *H*_e_ of all populations (0.53 vs. range: 0.61–0.76). Low genetic diversity, which was also observed with mtDNA and at the Y-chromosome, has earlier been attributed to a historical bottleneck (≤ 75 individuals between 1895 and 1930) [20].

**Fig 4:**
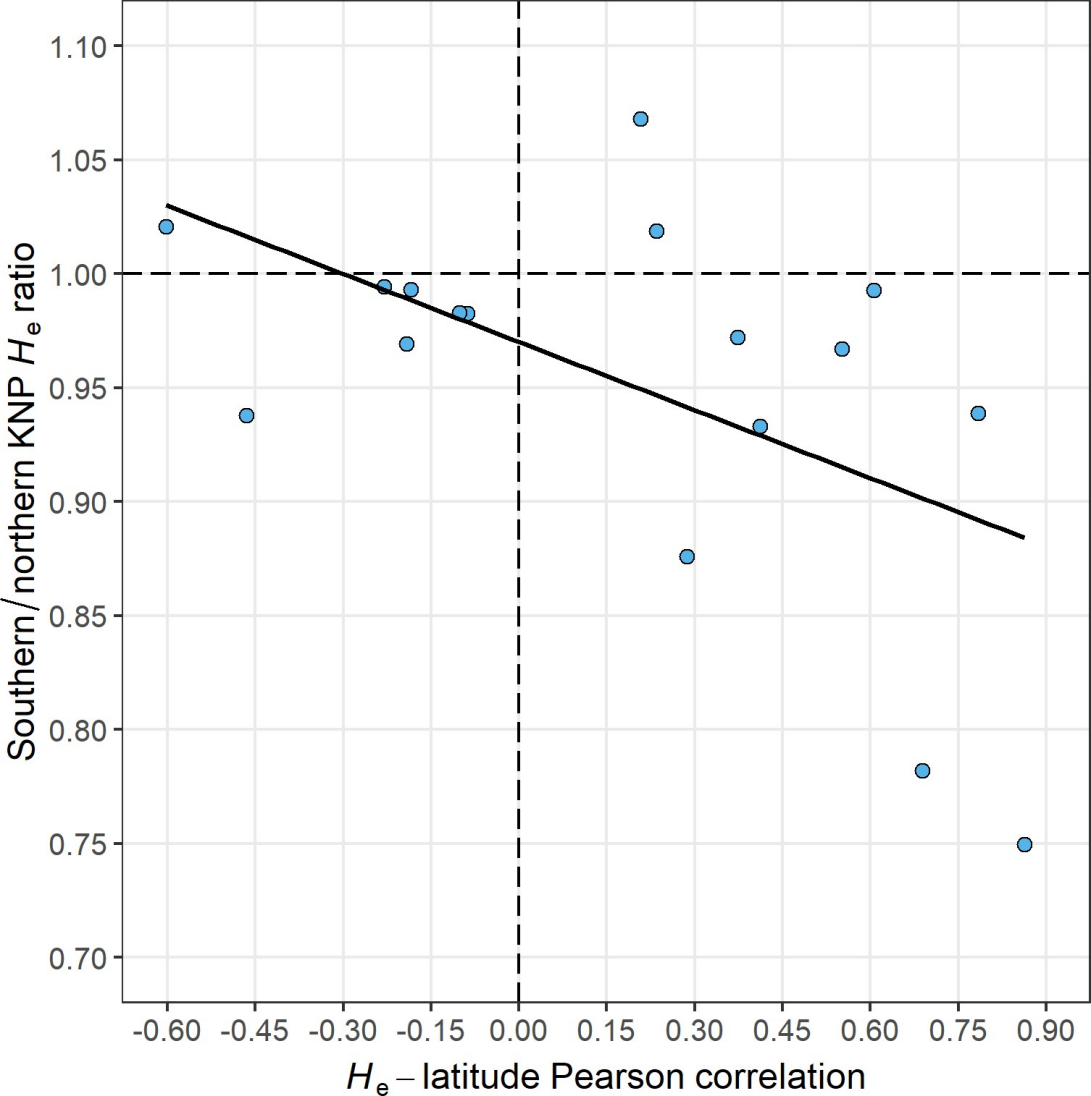
Correlation between per-locus southern/northern KNP *H*_e_ ratio and *H*_e_-latitude Pearson correlation.

There was a significant Spearman correlation between the per-locus southern/northern KNP *H*_e_ ratio on the one hand and the continent-wide Pearson correlation between *H*_e_ and latitude on the other (Spearman *ρ* = -0.55, *P* = 0.021, *N* loci = 17, Fig 4; Spearman *ρ* = -0.51, *P* = 0.025, *N* loci = 19, when also including *ABS010* and *AGLA293*, S4 Fig). This indicates that selective sweeps in southern KNP caused by the introduction of BTB around 1960 [21, 22] were correlated with historical selective sweeps (since > 100 years before sampling) across Africa.

### Linkage disequilibrium

The total fraction of locus pairs with positive *r*_LD_ across populations and linked loci was 0.66, which was higher than expected under random association (LD Test 1; Wilson score 95% CI: 0.53–0.77, *N* = 53, randomizing genotypes: *P* = 0.015, randomizing allele assignments: *P* = 0.0038; S7 Table), although these locus pairs were not observed at significantly low latitudes (LD Test 2; *P* > 0.35; however, this test had low statistical power—see Methods section). The fraction with positive *r*_LD_ was > 0.5 at all five linked locus pairs (Sign test: *P* = 0.063) but was particularly high for interlocus distances ≥ 19 Mb (three locus pairs: 0.68, *N* = 40; randomizing genotypes: *P* = 0.028, randomizing allele assignments: *P* = 0.0052). Surprisingly, the fraction with positive *r*_LD_ was also relatively high in East Africa (four locus pairs: 0.74, *N* = 19; randomizing genotypes: *P* = 0.031, randomizing allele assignments: *P* = 0.0081). Both among randomized genotypes at the former five locus pairs and among observed genotypes at the remaining unlinked locus pairs, the total fraction with positive *r*_LD_ was very close to the expected value of 0.5 (randomized genotypes: 0.500, *N* = 2,422,396; remaining loci: 0.501, *N* = 1095).

On average, the four locus pairs that earlier showed significant LD in KNP based on the non-pooled microsatellite alleles also showed significantly positive *r*_LD_ values with the p-MDTA alleles, particularly the three neighbouring pairs *BM1824*-*BM3205*, *BM1824*-*CSMM019* and *CSMM019*-*BM3205* (LD Test 3; all pairs: average *r*_LD_ = 0.203, exact *P* = 0.019; neighbouring pairs: average *r*_LD_ = 0.284, exact *P* = 0.010; Table 3). This indicates that LD was not only caused by physical linkage but also by selection. The *r*_LD_ values based on random allele assignments were close to zero on average (average *r*_LD_ = 0.035, Table 3), while no meaningful correlation in *r*_LD_ was observed between the neighbouring locus pairs (Pearson *r* ≤ 0.12). The latter two observations indicate that, in the absence of selection, strong LD based on non-pooled alleles does not necessarily result in strong LD based on pooled alleles, and that *r*_LD_ values at neighbouring locus pairs can be statistically independent. The three LD tests based on random allele assignments combined indicate highly significant LD due to selection (Test 1: *Z* = 2.48, after excluding KNP for independence with Test 3; Test 2: *Z* = -0.31; Test 3: *Z* = 2.35; Z-transform test: combined Z = 2.61, combined *P* = 0.0090) [64].

## Discussion

We observed continent-wide frequency clines of p-MDTA alleles with decreasing frequencies in a northerly direction, which could be attributed to selection (*P* = 0.025). Further, the multilocus-*H*_e_ cline (*P* = 0.029) and the correlation between short- and long-term decrease in per-locus *H*_e_ (*P* = 0.023) suggests that via genetic hitchhiking genome-wide selection has had a negative effect on population-level genetic diversity. Although no significant LD cline was observed (*P* > 0.35), both the high fraction of locus pairs with positive *r*_LD_ and the high average *r*_LD_ in KNP could be explained by selection (*P* ≤ 0.015 and *P* = 0.019, respectively). Positive LD is indicative of genome-wide selection resulting in increased frequencies of haplotypes consisting of multiple deleterious alleles (relative to linkage equilibrium). Relatively high p-MDTA allele frequencies (*P* = 0.083) and significant positive LD (*P* ≤ 0.031) in East Africa indicate that the male-deleterious alleles are active across the entire latitudinal range.

Our results may be explained by continent-wide and genome-wide selection for male-deleterious traits, indicative of a high genetic load, with selection strength decreasing from south to north. The only exception is the population in HiP, where male-deleterious alleles appear to be under negative selection (relatively low p-MDTA allele frequencies). The selection pressures in East and southern Africa must be high to prevent destruction of the allele-frequency clines and haplotypes by LD decay. Three statistically independent results, i.e., the allele-frequency clines, the short vs. long-term *H*_e_-decrease correlation and LD, two of which did not rely on interlaboratory allele alignment (short vs. long-term *H*_e_-decrease and LD in KNP), provide strong support for earlier reported observations of genome-wide selection (S1 Table) [20–22].

### Allele-frequency clines

Common explanations for allele-frequency clines due to selection assume the advance of a favourable mutation (paradoxically in our case male-deleterious alleles), an environmental gradient, or a combination of the two [28, 65]. The advance of a favourable mutation assumes South Africa as the place of origin of the male-deleterious alleles. However, we consider it unlikely that all male-deleterious alleles originated in a relatively small area. The presence of a linear environmental gradient seems an unlikely explanation considering the wide range of habitats and climates in the sampled region that overall are non-linearly distributed. To our knowledge, there is no linear disease gradient in this region either. Further, the advance of a favourable mutation, an environmental gradient or a disease gradient would not explain the high frequencies of the male-deleterious alleles, which normally are under negative selection [66]. It is also implausible that the allele-frequency clines are a result of the selective agent being present only in the most southern part of the range (e.g., South Africa) considering the occurrence of positive LD in East Africa. LD decay is expected to be very fast in the absence of selection (6–31% per generation at the locus pairs analysed in East Africa), considering the chromosomal distances involved.

Instead of the aforementioned explanations, we posit that the continent-wide allele-frequency clines are caused by the same sex-ratio meiotic gene-drive system that earlier has been hypothesized to be the ultimate selective agent in KNP and HiP. We do so in light of our earlier work showing that both male-deleterious alleles and gene-drive system interact with diseases, such as BTB, and other environmental factors, such as droughts [20–22]. We hypothesize that there is a similar continent-wide frequency cline of gene-drive alleles, also with decreasing frequencies in a northerly direction, considering that in KNP MDTA allele-frequency clines co-occurred with an allele-frequency cline of a Y-chromosomal haplotype linked to a Y-suppressor gene (Y haplotype 557) [22]. Our explanation for the allele-frequency clines implies that frequencies of male-deleterious alleles on the autosomes are maintained at specific equilibrium values in each population depending on the local frequencies of the gene-drive alleles on the sex chromosomes. This would require some form of balancing selection (which does not preclude periods of positive or negative selection such as observed in KNP and HiP, respectively [20–22]).

### Genetic hitchhiking

The increase in frequency of male-deleterious alleles to equilibrium can be described as a “partial selective sweep” [30], with each sweep causing a hitchhiking effect and decreased *H*_e_ at linked loci, such as observed for the microsatellites in this study. Although selective sweeps are generally observed with directional selection, they can also occur during the early phase of balancing selection [67]. In a recent study in KNP and HiP, decreased *H*_e_ at neighbouring loci *BMS1617* and *IFNG* relative to other nearby loci has been attributed to a selective sweep, providing additional support for this scenario [68]. The relatively low microsatellite *H*_e_ in southern Africa is unlikely to be caused by low effective population size considering the correlation between short-term hitchhiking effects in southern KNP and long-term hitchhiking effects across Africa, and considering that mtDNA nucleotide diversity (*π*) is actually considerably higher in southern Africa than in East Africa (D-loop: 0.047 vs. 0.036; complete mitogenome: 0.0048 vs. 0.0035) [69, 70]. The observed correlation between short-term and long-term hitchhiking effects indicates that environmental stressors such as drought and diseases have been consistently acting as selective agents for long periods of time.

Interestingly, pairwise sequentially Markovian coalescent (PSMC) models based on whole-genome sequences indicate relatively high instantaneous coalescence rates since ~20,000 years ago in South Africa, but not in Kenya [71, 72]. According to population genetic theory, high coalescence rates are associated with low heterozygosity because heterozygosity is proportional to the coalescent time [73]. Although the authors attributed the high coalescence rates in South Africa to a low effective population size, they may also be a signal of selection and hitchhiking effects since the introduction of the aforementioned sex-ratio meiotic gene-drive system [74, 75]. If so, then we predict low heterozygosity to be genome-region specific (i.e., near male-deleterious alleles) and not genome wide. It is relevant to note here that multilocus heterozygosity at microsatellites is a reliable indicator of genome-wide heterozygosity, considering that relative differences in multilocus heterozygosity among South African populations were similar between genome-wide sequence data and microsatellites (observed heterozygosity genome-wide sequence data: KNP = 0.00324, HiP = 0.00261, Addo NP = 0.00222; multilocus *H*_e_ microsatellites: KNP = 0.686 [38 loci], HiP = 0.581 [38 loci], Addo NP = 0.447 [18 loci]; S8 Table) [20, 22, 68, 71, 76, 77].

### Linkage disequilibrium

Positive LD at linked loci indicates increased frequencies of deleterious-allele haplotypes relative to linkage equilibrium. We detected long-distance LD at genomic distances ≥ 5.9 Mb at four locus pairs; these pairs occur on chromosomes 1 and 3 in cattle. Long-distance LD appears to occur genome-wide considering that significant LD has earlier been observed in KNP at genomic distances of 7.7–17.9 Mb at three locus pairs (six loci) in a 34 Mb region, occurring on chromosome 5 in cattle (*D*’ = 0.28–0.43; i.e., LD is at 28–43% of its maximum possible value) [54, 68]. The observation of long-distance LD in KNP at chromosomal distances ≥ 5.9 Mb is surprising, especially considering that high haploid and autosomal diversity indicate a large historical effective population size for the KNP buffalo (34 mitochondrial D-loop haplotypes with gene diversity *H* = 0.94; 15 Y-chromosomal haplotypes with *H* = 0.74; whole-genome sequencing: high genome-wide observed heterozygosity compared to other mammals: *H* = 0.00324, low median inbreeding coefficient: *f* = 0.00060) [21, 24, 71, 78]. According to population genetic theory, a large effective population size limits long-distance LD because of increased LD decay with chromosomal distance [62].

LD in buffalo populations extends across much larger chromosomal distances than in other natural mammal populations that we are aware of for which LD decay has been estimated. In chimpanzees (*Pan troglodytes*) and bonobos (*Pan paniscus*) LD extends to a distance of ~0.15 Mb, in Arizona wild mice (*Mus musculus domesticus*) to a distance of ~0.2 Mb and in Iberian wild boar (*Sus scrofa*) to a distance of ~0.5 Mb. In gray wolf (*Canus lupus*) and coyote (*Canus latrans*) LD extends to a distance of ~5 Mb, even in small or bottlenecked populations apart from the wolf population of Isle Royale, which consisted of just 10–30 individuals [79–84]. Further, the half-length of LD (the distance at which LD is 50% of its maximal value) in two isolated Canadian populations of bighorn sheep (*Ovis canadensis*) is only 25%-40% of that in KNP buffalo, despite their small size of less than 200 individuals each (4.6–7.5 Mb vs. ~20 Mb at chromosome 5 in buffalo) [68, 85, 86].

LD between distant loci in large outbreeding buffalo populations, despite fast LD decay due to recombination (~21–31% per generation for three out of six locus pairs), indicates strong selection pressures. Simulation studies, however, indicate that multilocus selection may lower the minimum selection pressure necessary for a given level of LD and may slow down LD decay in a multilocus cline (although even then selection is probably still relatively strong) [31, 87]. Further, allele frequency differences between male and female gametes due to sex-specific selection, as hypothesized in KNP based on genetic data from male and female calves [21], may have resulted in admixture LD between distant loci, especially when selection is strong [32].

### Genetic load

A substantial number of high-frequency deleterious alleles of seemingly large effect [21, 22], with many co-occurring in haplotypes (relative to what one would expect if no linkage existed among these alleles), indicates a high genetic load. Most populations of African buffalo, however, seem to have been large in the evolutionary past (long-term *N*_e_ of *S*. *c*. *caffer*: ~ 48,000) [71] and to be stable after their recovery from the rinderpest pandemic of 1889–1895 that decimated buffalo populations across the whole of Africa [33, 88]. At face value, this seems to support the view, advocated by some population geneticists, that genetic load and genetic diversity in general play a smaller role in ecology than one might expect [6, 89].

To bolster the significance of ecological versus genetic factors in explaining the stability of buffalo populations, we suggest the following five non-mutually exclusive explanations:

1. Male-deleterious alleles are epigenetically suppressed in a large fraction of animals [20, 21].
2. Net-deleterious effects on female health are diminished or even prevented by male-deleterious alleles or haplotypes with negative phenotypic effects in males but positive phenotypic effects in females [22].
3. Negative phenotypic effects only become evident in stressful periods, such as those caused by droughts and disease outbreaks. For example, in 2001 average body condition was lower in southern KNP, a region characterized by relatively high frequencies of MDTA alleles, than in northern KNP, but only significantly so at the end of the dry season [90].
4. Selection is mostly soft, where selective death due to male-deleterious alleles would otherwise be replaced by nonselective death [6].
5. Interspecific competition is limited, thereby minimizing the ecological effects of genetic load [6]. A recent review has failed to find conclusive evidence for interspecific competition between African buffalo and other large mammals [91], which may be due to ecological separation [92].

## Conclusions

Our analyses and results reveal that selection resulted in a continent-wide and genome-wide distribution of high-frequency male-deleterious alleles in the African buffalo, with many co-occurring in haplotypes (relative to what one would expect if no linkage existed among these alleles), and decreased population-level genetic diversity via genetic hitchhiking. Since most populations appear to be stable, this suggests that natural populations of mammals, depending on their genetic background, can withstand a high genetic load. Nevertheless, we expect that a high genetic load makes many buffalo populations vulnerable to environmental stressors such as droughts and disease outbreaks. Since buffalo play an important role in the maintenance and transmission of a variety of economically important livestock diseases, which may well have been augmented by a high genetic load, particularly in southern Africa, our results have relevance to livestock husbandry in areas were cattle occur in close proximity to buffalo herds.

## Supporting information

S1 Text Allele size standardization. (DOCX)

S1 Table Earlier reported associations of microsatellite alleles with low body condition and bovine tuberculosis (BTB) infection risk. (DOCX)

S2 Table Allele size standardization. (DOCX)

S3 Table Overview of alleles selected for analysis. (CSV)

S4 Table p-MDTA allele frequencies per population. (CSV)

S5 Table Per-locus Pearson correlation between ‘p-MDTA minus p-wildtype’ allele frequency difference and latitude, and between *H*_e_ and latitude. (CSV)

S6 Table *H*_e_ per locus per population. (CSV)

S7 Table Linkage disequilibrium per population. (CSV)

S8 Table *H*_e_ per locus in KNP, HiP and Addo NP. (CSV)

S1 Fig Increase of pairwise *F*_ST_ with geographic distance. (DOCX)

S2 Fig Multilocus-*H*_e_ cline based on twelve microsatellites per locality. (DOCX)

S3 Fig Multilocus-*H*_e_ cline based on seven microsatellites analysed in both East and southern Africa (microsatellite sets A, B and D) plus *ABS010* and *AGLA293*. (DOCX)

S4 Fig Correlation between per-locus southern/northern KNP *H*_e_ ratio and *H*_e_-latitude Pearson correlation, based on 19 microsatellites. (DOCX)
