# Supplementary material for: A continent-wide high genetic load in African buffalo revealed by clines in the frequency of deleterious alleles, genetic hitchhiking and linkage disequilibrium
Source: PLoS One. 2021 Dec 9;16(12):e0259685. doi: 10.1371/journal.pone.0259685 (PMC8659316; doi:10.1371/journal.pone.0259685)
Supplement: S1 Text — (DOCX) [file pone.0259685.s001.docx]

**Text S1: Allele size standardization**

Microsatellite sets B and C were both available for Masai Mara GR, Lake Nakuru NP and Amboseli NP (total number of genotyped individuals: *N*_B_ = 65, *N*_C_ = 70). Microsatellite sets A and B were both available for KNP (*N*_A_ = 459, *N*_B_ = 38). Microsatellite sets A and D were both available for KNP and HiP (*N*_A_ = 860, *N*_D_ = 48). In the latter comparison, we included both northern and southern KNP, although set D was only analysed in northern KNP, to get more accurate allele frequency estimates for set A. Including both northern and southern KNP was justified because of the very small *F*_ST_ value between these localities (*F*_ST_ = 0.0034, 95% CI = 0.0023, 0.0045). In all aforementioned set comparisons, size standardization was based on the allele frequencies of the pooled samples. A detailed depiction of the allele size standardization is given in Table S1. *F*_ST_ values and G-statistics derived *P* values (9999 randomizations) were estimated with the Genalex add-in for Excel (version 6.503).

Microsatellite sets E and F were only available for one locality each; Serengeti NP and Caprivi Strip, respectively. Standardization of these two sets was only possible by comparison with localities in southern Africa: Niassa Reserve (Niassa analysed with set D, distance to Serengeti NP: ± 1150 km; *N*_D_ = 20, *N*_E_ = 49) in case of set E and the pooled samples of northern and southern KNP in case of set F (KNP analysed with set A, distance to Caprivi Strip: ± 1100 km; *N*_A_ = 459, *N*_F_ = 134).

Size standardization of sets B and D against set A was unambiguous (Figures 1 and 2), even when allele size difference was ≥ 8 bp (*BM3517* and *INRA006*, Table 1). Allele frequencies in sets B and D strongly correlated with those in set A (*r* per individual locus ≥ 0.88, *F*_ST_ per individual locus ≤ 0.012). No allele in set B and only one allele in set D (frequency = 0.043) observed more than twice were not observed in set A (more unique alleles vice versa because of the relatively small sample sizes of sets B and D). No significant differentiation was observed between sets, neither for individual loci after Bonferroni correction (α = 0.0042, uncorrected *P* ≥ 0.038), nor for all loci combined (set B: *P* = 0.12, *F*_ST_ = 0.005, set D: *P* = 0.21, *F*_ST_ = 0.004).


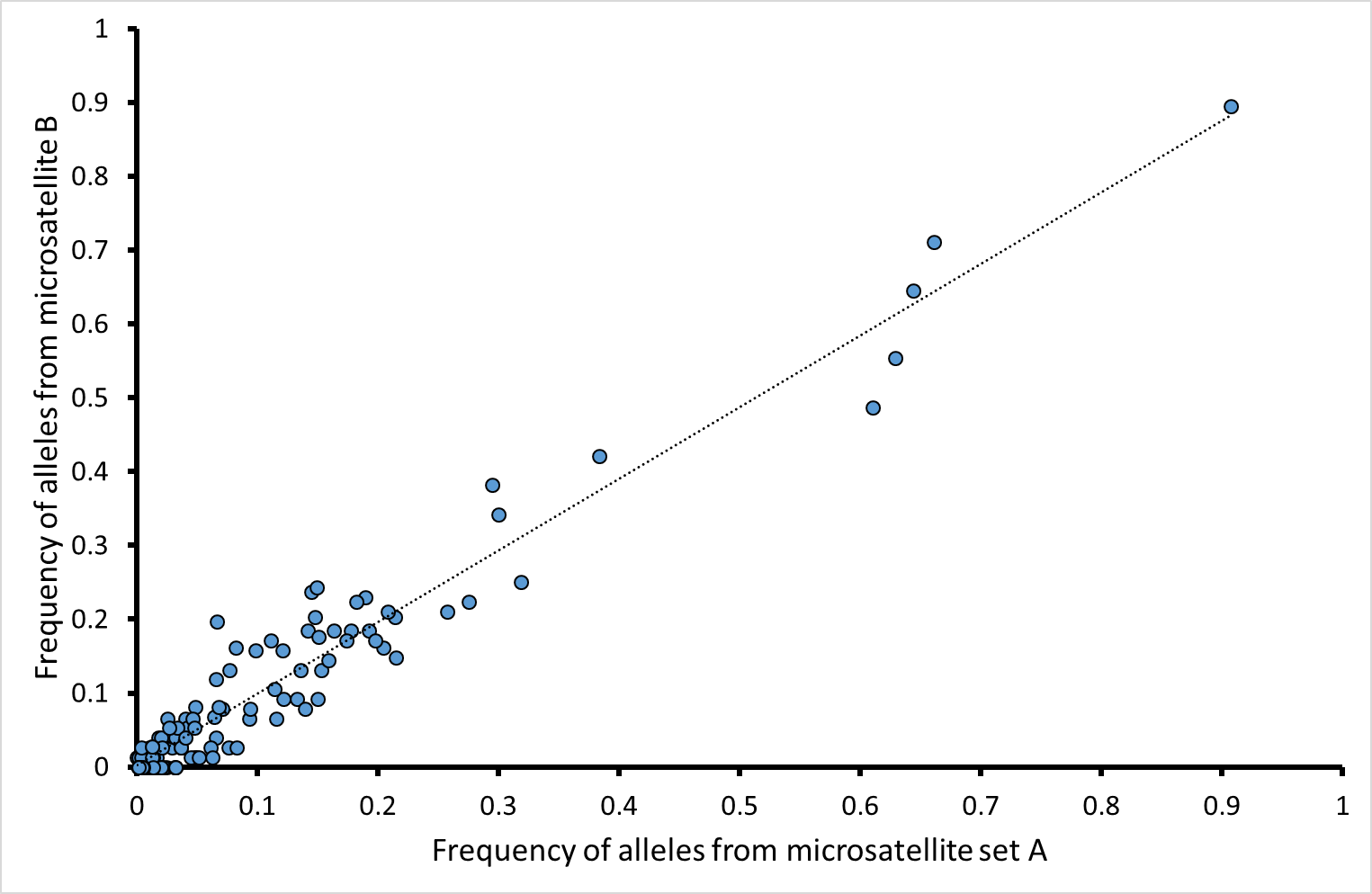


Figure 1: Size standardization of microsatellite set B against microsatellite set A

*N*_microsatellites_ = 12, *N*_alleles_ = 122, *N*_individuals_ = 497, Pearson *r* = 0.97, *F*_ST_ = 0.005, *P*_FST_ = 0.12


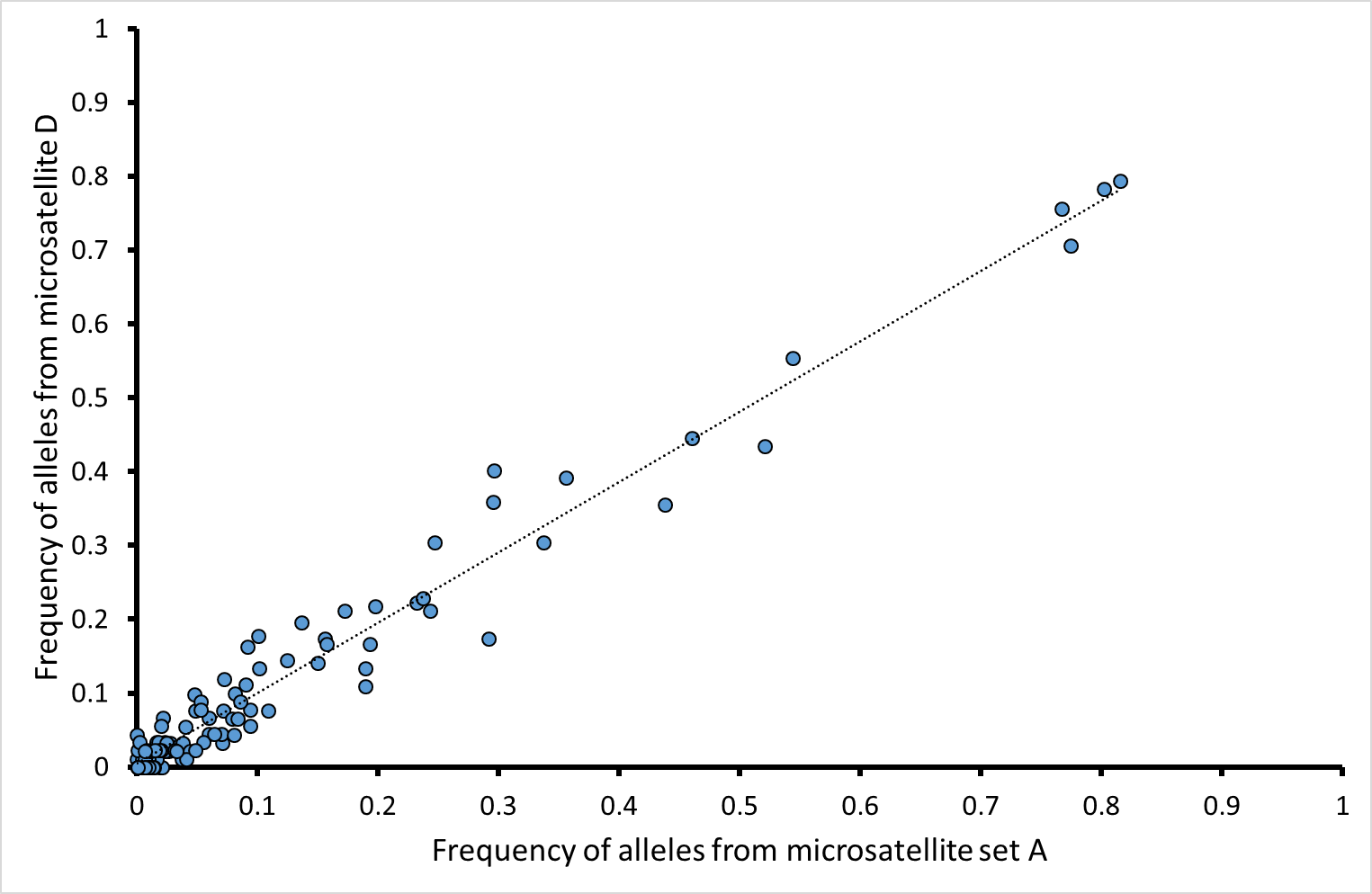


Figure 2: Size standardization of microsatellite set D against microsatellite set A

*N*_microsatellites_ = 12, *N*_alleles_ = 108, *N*_individuals_ = 906, Pearson *r* = 0.98, *F*_ST_ = 0.004, *P*_FST_ = 0.21

Table 1: Size standardization of *BM3517* and *INRA006*, set B against set A

*BM3517*: *r* = 0.998, *F*_ST_ = 0.002, *P* = 0.606; *INRA006*: *r* = 0.956, *F*_ST_ = 0.012, *P* = 0.038; *N*_A_ = 459, *N*_B_ = 38.

| *BM3517* allele size (A/B) | Frequency set A | Frequency set B |
| --- | --- | --- |
| 84/92 | 0.006 | 0.013 |
| 86/94 | 0.093 | 0.066 |
| 88/- | 0.009 | 0 |
| 90/98 | 0.029 | 0.026 |
| 92/100 | 0.661 | 0.711 |
| 94/102 | 0.178 | 0.184 |
| 96/- | 0.024 | 0 |
| *INRA006* allele size (A/B) | Frequency set A | Frequency set B |
| 105/- | 0.001 | 0 |
| 107/- | 0.004 | 0 |
| 109/102 | 0.149 | 0.243 |
| 111/122 | 0.062 | 0.014 |
| 113/124 | 0.610 | 0.486 |
| 115/126 | 0.148 | 0.203 |
| 117/128 | 0.012 | 0.027 |
| 119/130 | 0.012 | 0.027 |
| 127/- | 0.001 | 0 |

At three loci in set C (*BM0719*, *DIK020* and *ILSTS026*; Table 2), we had to assume that one allele was erroneously called, because its two neighbouring alleles, which differed by two repeats, differed by only one repeat in set B. Erroneous allele calling is far more likely in set C than in set B, considering the aforementioned unambiguous size standardization of set B against set A. At loci *BM0719* and *DIK020*, the erroneously called alleles were observed only once in all the populations analysed with set C (*BM0719*: allele with original size 145 in Nairobi NP, *DIK020*: allele with original size 197 in Laikipia NP). However, the erroneously called allele at locus *ILSTS026* was observed in six out of ten populations at an average frequency of 0.10 (allele with original size 152). This allele could correspond to two alleles in set B; one male-deleterious-trait-associated and the other non-associated. We conservatively chose to let it correspond with the male-deleterious-trait-associated allele because this resulted in a more positive Pearson *r* when correlating allele frequencies against latitude (i.e., less significant allele-frequency cline). Further, allele size calling at locus *ILSTS026* did not influence linkage disequilibrium estimates because neighbouring locus *INRA006* was not included in set C. Despite the relatively high frequencies of the supposedly erroneous called allele, frequencies at locus *ILSTS026* correlated strongly between sets B and C.

Table 2: Size standardization of *BM0719*, *ILSTS026* and *DIK020*, set C against set B

*BM0719*: *r* = 0.874, *F*_ST_ = 0.005, *P* = 0.291; *ILSTS026*: *r* = 0.825, *F*_ST_ = 0.003, *P* = 0.522; *DIK020*: *r* = 0.626 (*r* = 0.861 without allele 188), *F*_ST_ = 0.011, *P* = 0.003; *N*_B_ = 65, *N*_C_ = 70.

| *BM0719* allele size (A/B/C) | Frequency set B | Frequency set C |
| --- | --- | --- |
| 132/133/133 | 0.023 | 0.014 |
| 134/-/135 | 0 | 0.007 |
| 136/137/137 | 0.086 | 0.079 |
| 138/139/139 | 0.023 | 0.021 |
| 140/141/141 | 0.125 | 0.093 |
| 142/143/143 | 0.070 | 0.064 |
| 144/145/147 | 0.125 | 0.093 |
| 146/147/149 | 0.023 | 0.021 |
| 148/150/151 | 0.195 | 0.293 |
| 150/152/153 | 0.023 | 0.057 |
| 152/154/155 | 0.023 | 0.029 |
| 154/156/157 | 0.125 | 0.129 |
| 156/158/159 | 0.156 | 0.100 |
| *ILSTS026* allele size (A/B/C) | Frequency set B | Frequency set C |
| 143/141/144 | 0.123 | 0.143 |
| 147/145/148 | 0.200 | 0.136 |
| 149/147/150 | 0.038 | 0.029 |
| ?/-/152 | 0 | 0.014 |
| 151/149/154 | 0.069 | 0.107 |
| 153/152/156 | 0.146 | 0.114 |
| 155/154/158 | 0.208 | 0.186 |
| 157/156/160 | 0.116 | 0.114 |
| 161/160/164 | 0.069 | 0.129 |
| 163/162/166 | 0.031 | 0.029 |
| *DIK020* allele size (A/B/C) | Frequency set B | Frequency set C |
| 166/169/169 | 0.065 | 0.065 |
| 168/171/171 | 0.016 | 0.029 |
| 170/173/173 | 0.145 | 0.232 |
| 172/175/175 | 0.105 | 0.101 |
| 174/177/177 | 0.040 | 0.051 |
| 176/179/179 | 0.081 | 0.109 |
| 178/181/181 | 0.024 | 0.014 |
| 180/-/183 | 0 | 0.014 |
| 6-7 bp gap in sets B and C |  |  |
| 186/188/189 | 0.073 | 0.029 |
| 188/190/191 | 0.218 | 0.072 |
| 190/192/193 | 0.056 | 0.130 |
| 8 bp gap in sets B and C |  |  |
| 196/-/201 | 0 | 0.007 |
| 198/200/203 | 0.073 | 0.087 |
| 200/202/205 | 0.065 | 0.036 |
| 6 bp gap in sets B and C |  |  |
| 206/208/211 | 0.032 | 0.022 |
| 28 bp gap in set B |  |  |
| 234/236/- | 0.008 | 0 |

No significant differentiation was observed between sets B and C when considering all ten loci together (*P* = 0.063, *F*_ST_ = 0.005; Figure 3). Except for locus *DIK020*, no significant differentiation was observed at individual loci after Bonferroni correction (α = 0.005, *DIK020*: uncorrected *P* = 0.0027, other loci: uncorrected *P* ≥ 0.048). High significance at locus *DIK020* could be attributed to a single allele with a relatively low frequency in set C (frequency set B = 0.22, frequency set C = 0.07; *r* = 0.63 when allele is included, *r* = 0.86 when allele is excluded; Table 2). Because significance could be attributed to a single allele and because *F*_ST_ was low regardless (*F*_ST_= 0.011) we do not consider this a meaningful deviation. Except for locus *DIK020*, allele frequencies correlated strongly between sets B and C (*r* per individual locus ≥ 0.73, *F*_ST_ per individual locus ≤ 0.007). Only eight alleles (out of 109) observed more than twice in one set were not observed in the other (maximum frequency of these eight alleles = 0.089).


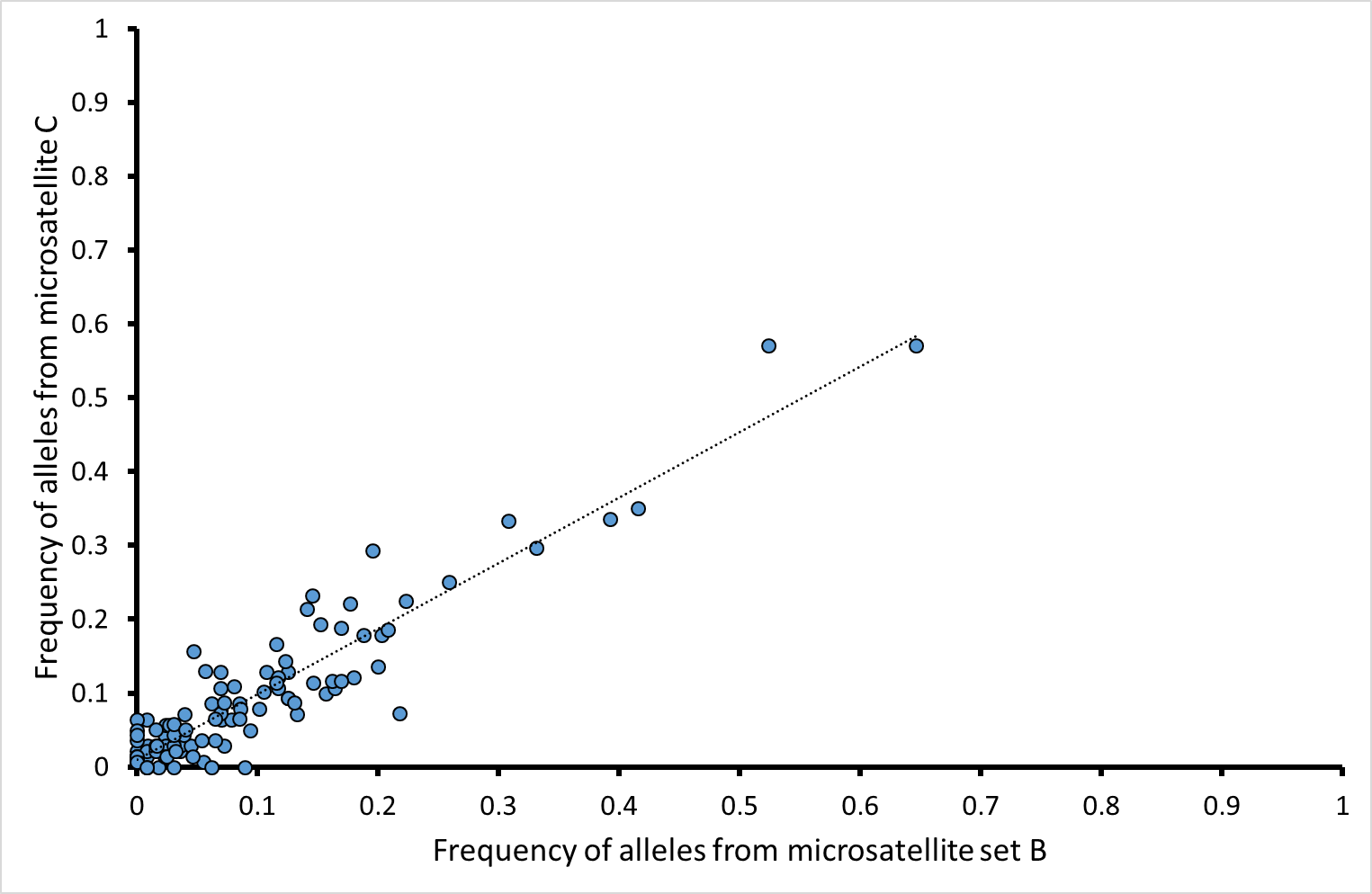


Figure 3: Size standardization of microsatellite set C against microsatellite set B

*N*_microsatellites_ = 10, *N*_alleles_ = 109, *N*_individuals_ = 135, Pearson *r* = 0.93, *F*_ST_ = 0.005, *P*_FST_ = 0.063

Allele frequencies of the three loci in set D were positively correlated with those in set E, with size shifts ≤ 2 bp (*BM1824*: *r* = 0.39, *F*_ST_ = 0.031, *P* = 0.003; *ETH010*: *r* = 0.88, *F*_ST_ = 0.058, *P* = 0.0005; *SPS115*: *r* = 0.78, *F*_ST_ = 0.007, *P* = 0.62; Table 3). Significant differentiation was observed at loci *BM1824* and *ETH010*. However, no significant differentiation was observed at locus *BM1824* when compared with the pooled sample of Mana Pools NP, Nyakasanga, Gorongosa NP and Marromeu GR, which are located 730 km further south (*r* = 0.78, *F*_ST_ = 0.012, *P* = 0.065). Significant differentiation at locus *ETH010* could be attributed due to a unique high-frequency allele in Serengeti NP (frequency = 0.23) not observed in southern Africa (without this allele: *r* = 0.92). The size standardization of the two loci in set F against set A was unambiguous, characterized by high allele-frequency correlations and low *F*_ST_ values, despite the fact these sets were applied in different populations more than 1000 km apart (*BM3517*: *r* = 0.99, *F*_ST_ = 0.004, *P* = 0.023; *TGLA057*: *r* = 0.89, *F*_ST_ = 0.011, *P* < 0.001; Table 4).

Table 3: Size standardization of *BM0719*, *ILSTS026* and *DIK020*, set E against set D

*BM1824*: *r* = 0.394 (*r* = 0.781 with the pooled sample of Mana Pools NP, Nyakasanga, Gorongosa NP and Marromeu GR), *F*_ST_ = 0.031, *P* = 0.003; *ETH010*: *r* = 0.883, *F*_ST_ = 0.058, *P* = 0.0005; *SPS115*: *r* = 0.781, *F*_ST_ = 0.007, *P* = 0.620; *N*_E_ = 49, *N*_D_ = 40

| *BM1824* allele size (A/E/D) | Frequency Serengeti NP (set E) | Niassa Reserve (set D) |
| --- | --- | --- |
| 169/167/169 | 0.216 | 0.211 (0.145) |
| 175/175/175 | 0.136 | 0.079 (0.092) |
| 177/177/- | 0.011 | 0 (0) |
| 179/179/179 | 0.011 | 0 (0.013) |
| 181/181/181 | 0.273 | 0.105 (0.250) |
| 183/9183/183 | 0.091 | 0.105 (0.039) |
| 185/185/185 | 0.057 | 0.026 (.0.066) |
| 187/187/187 | 0.159 | 0.053 (0.053) |
| 189/189/189 | 0.011 | 0.053 (0.013) |
| 191/191/191 | 0.023 | 0.263 (0.066) |
| 193/-/193 | 0 | 0.053 (0.066) |
| 195/195/195 | 0.011 | 0.026 (0.105) |
| 197/-/197 | 0 | 0 (0.066) |
| 199/-/199 | 0.000 | 0.026 (0.026) |
| *ETH010* allele size (A/E/D) | Frequency set E | Frequency set D |
| 200/200/- | 0.010 | 0 |
| 202/202/- | 0.229 | 0 |
| 204/204/204 | 0.479 | 0.750 |
| 206/206/206 | 0.271 | 0.250 |
| 208/208/208 | 0.010 | 0 |
| *SPS115* allele size (A/E/D) | Frequency set E | Frequency set D |
| 223/-/227 | 0 | 0.075 |
| 227/228/231 | 0.053 | 0.025 |
| 229/230/233 | 0.128 | 0.075 |
| 231/232/237 | 0.117 | 0.075 |
| 233/234/239 | 0.085 | 0.100 |
| 237/236/- | 0.053 | 0 |
| 239/238/245 | 0.255 | 0.275 |
| 241/240/247 | 0.064 | 0.150 |
| 243/242/249 | 0.074 | 0.100 |
| 245/244/251 | 0.128 | 0.100 |
| 247/246/- | 0.043 | 0 |
| 249/-/255 | 0 | 0.025 |

Table 4: Size standardization of *BM3517* and *TGLA057*, set F against set A

*BM3517*: *r* = 0.988, *F*_ST_ = 0.004, *P* = 0.023; *TGLA057*: *r* = 0.887, *F*_ST_ = 0.011, *P* = 0.0001; *N*_A_ = 459, *N*_F_ = 134

| *BM3517* allele size (A/F) | Frequency KNP (set A) | Frequency Caprivi Strip (set F) |
| --- | --- | --- |
| 84/- | 0.006 | 0 |
| 86/91 | 0.093 | 0.112 |
| 88/93 | 0.009 | 0.011 |
| 90/95 | 0.029 | 0.078 |
| 92/97 | 0.661 | 0.690 |
| 94/99 | 0.178 | 0.108 |
| 96/- | 0.024 | 0 |
| *TGLA057* allele size (A/F) | Frequency set A | Frequency set B |
| 75/- | 0.002 | 0 |
| 79/- | 0.001 | 0 |
| 83/- | 0.001 | 0 |
| 89/- | 0.051 | 0 |
| 91/93 | 0.001 | 0.004 |
| 93/95 | 0.275 | 0.149 |
| 95/97 | 0.257 | 0.235 |
| 97/99 | 0.295 | 0.388 |
| 99/101 | 0.076 | 0.086 |
| 101/103 | 0.040 | 0.060 |
| 103/105 | 0 | 0.078 |
