# Supplementary material for: A continent-wide high genetic load in African buffalo revealed by clines in the frequency of deleterious alleles, genetic hitchhiking and linkage disequilibrium
Source: PLoS One. 2021 Dec 9;16(12):e0259685. doi: 10.1371/journal.pone.0259685 (PMC8659316; doi:10.1371/journal.pone.0259685)
Supplement: S1 Table — (DOCX) [file pone.0259685.s002.docx]

**Table S1**: Earlier reported associations of microsatellite alleles with low body condition and bovine tuberculosis (BTB) infection risk indicative of linkage to male-deleterious alleles

| Direct associations with low body condition and BTB | |
| --- | --- |
| Neg. corr. between sexes in body-condition-dependent allele freq. difference (sexual antagonism) | KNP: *P* = 0.0024, Fig. 3 [1] |
| Largest sex. antagonistic body-condition-dependent difference in heterozygosity for the three most frequent alleles per locus | KNP: *P* = 0.027, Fig. 4 [1] |
| Low body condition most frequent among homozygotes for the most frequent allele per locus | KNP: *P* < 0.033, Fig. 2 [1] |
| Genetic associations with body condition and BTB mainly among animals born after multi-year wet periods (epigenetic effects) | KNP: *P =* 0.010, Table 1 [2]; HiP: *P =* 0.068, Table 4 [3] |
| Indirect associations with low body condition and BTB: signatures of selection | |
| Allele freq. clines between northern KNP (high body condition and BTB present) and southern KNP (low body condition and BTB absent): pos. selection in KNP | KNP: *P* = 0.00044, Fig. 5 [1] |
| Corr. between KNP-HiP allele freq. difference and strength of sexual antagonism: neg. selection in HiP | HiP: *P* = 0.00032, Fig. 2-3 [3] |
| Indirect associations with low body condition: correlations with multi-year pre-conception rainfall (proxy for parental body condition) | |
| Corr. between microsatellite diversity per year-cohort (KNP: heterozygosity, HiP: allele freq.) and multi-year pre-conception rainfall | KNP: *P* = 0.0015, Fig. S5 [1]; HiP: *P* = 0.00018, Fig. 4 [3] |

HiP: Hluhluwe-iMfolozi Park, KNP: Kruger National Park.

**Table S1** cont.:

| Other associations with pre-conception rainfall related to the sex-ratio meiotic gene-drive system: sex ratio and Y-chromosomal haplotypes | |
| --- | --- |
| Female- and male-biased sex ratio among dry- and wet-season conceptions, respectively | KNP: *P* = 0.025, Table 1, Fig. 2 [4] |
| Pos. corr. between sex ratio per year-cohort and multi-year pre-conception rainfall (female-biased after dry years) | KNP: *P* = 0.041, Fig. 4 [4]; HiP: *P* = 0.0026, Fig. 6 [3] |
| Y-haplotypes associated with dry or wet season conceptions | KNP: *P* = 0.00089, Fig. 1 [4] |
| Y-haplotype frequencies per year-cohort associated with dry or wet multi-year pre-conception periods | KNP: *P* < 0.0001, Fig. 4 [4]; HiP: *P* = 0.043; Fig. 5 [3] |

HiP: Hluhluwe-iMfolozi Park, KNP: Kruger National Park.

**References**

1. van Hooft P, Greyling BJ, Getz WM, van Helden PD, Zwaan BJ, Bastos ADS. Positive selection of deleterious alleles through interaction with a sex-ratio suppressor gene in African buffalo: a plausible new mechanism for a high frequency anomaly. PLoS ONE. 2014;9(11):e111778.

2. van Hooft P, Dougherty ER, Getz WM, Greyling BJ, Zwaan BJ, Bastos ADS. Genetic responsiveness of African buffalo to environmental stressors: a role for epigenetics in balancing autosomal and sex chromosome interactions? PLoS ONE. 2018;13(2):e0191481.

3. van Hooft P, Getz WM, Greyling BJ, Bastos ADS. A natural gene drive system influences bovine tuberculosis susceptibility in African buffalo: possible implications for disease management. PLoS ONE. 2019;14(9):e0221168.

4. van Hooft P, Prins HHT, Getz WM, Jolles AE, van Wieren SE, Greyling BJ, et al. Rainfall-driven sex-ratio genes in African buffalo suggested by correlations between Y-chromosomal haplotype frequencies and foetal sex ratio. BMC Evol Biol. 2010;10:10.1186/471-2148-10-106. doi: 10.1186/1471-2148-10-106. PubMed PMID: MEDLINE:20416038.
