# Supplementary material for: A continent-wide high genetic load in African buffalo revealed by clines in the frequency of deleterious alleles, genetic hitchhiking and linkage disequilibrium
Source: PLoS One. 2021 Dec 9;16(12):e0259685. doi: 10.1371/journal.pone.0259685 (PMC8659316; doi:10.1371/journal.pone.0259685)
Supplement: S2 Table — (DOCX) [file pone.0259685.s003.docx]

**Table S1**: Summary of allele size standardization

| **Set** |  | **1** | **2** | **3** | **4** | **5** | **6** | **7** | **8** | **9** | **10** | **11** | **12** | **13** | **14** | **15** | **16** | **17** | **Overall^a^** |
| --- | --- | --- | --- | --- | --- | --- | --- | --- | --- | --- | --- | --- | --- | --- | --- | --- | --- | --- | --- |
| **B** | #alleles | 7 | 4 |  |  | 9 | 7 |  | 6 | 11 | 19 | 10 | 15 |  | 14 | 11 | 9 |  | 122 |
|  | *r* | 0.998 | 0.998c |  |  | 0.956 | 0.962 |  | 0.986 | 0.979 | 0.876 | 0.946 | 0.974 |  | 0.939 | 0.877 | 0.909 |  | 0.970 |
|  | *F*_ST_ | 0.002 | 0.003 |  |  | 0.012 | 0.011 |  | 0.005 | 0.003 | 0.004 | 0.006 | 0.003 |  | 0.003 | 0.003 | 0.005 |  | 0.005 |
|  | *P* value | 0.606 | 0.418 |  |  | 0.038 | 0.046 |  | 0.204 | 0.571 | 0.330 | 0.188 | 0.711 |  | 0.538 | 0.549 | 0.363 |  | 0.116 |
| **C** | #alleles | 6 | 13 |  |  |  | 6 |  |  | 13 | 16 | 11 | 13 |  | 13 | 9 | 9 |  | 109 |
|  | *r* | 0.974 | 0.969 |  |  |  | 0.994 |  |  | 0.733^b^ | 0.626^c^ | 0.962 | 0.874 |  | 0.912 | 0.825 | 0.944 |  | 0.932 |
|  | *F*_ST_ | 0.005 | 0.007 |  |  |  | 0.003 |  |  | 0.007 | 0.011 | 0.002 | 0.005 |  | 0.004 | 0.003 | 0.003 |  | 0.005 |
|  | *P* value | 0.197 | 0.119 |  |  |  | 0.401 |  |  | 0.048 | 0.003 | 0.760 | 0.291 |  | 0.428 | 0.522 | 0.566 |  | 0.063 |
| **D** | #alleles |  | 4 | 3 | 3 | 9 | 8 | 4 | 8 | 12 | 19 |  |  | 14 |  | 11 |  | 13 | 108 |
|  | *r* |  | 0.9996 | 0.9995 | 0.9994 | 0.983 | 0.995 | 0.969 | 0.958 | 0.927 | 0.966 |  |  | 0.918 |  | 0.974 |  | 0.903 | 0.983^d^ |
|  | *F*_ST_ |  | 0.001 | 0.000 | 0.001 | 0.002 | 0.005 | 0.004 | 0.008 | 0.008 | 0.005 |  |  | 0.003 |  | 0.003 |  | 0.003 | 0.004 |
|  | *P* value |  | 0.755 | 0.731 | 0.686 | 0.604 | 0.198 | 0.305 | 0.060 | 0.043 | 0.205 |  |  | 0.559 |  | 0.448 |  | 0.347 | 0.214 |
|  | #alleles |  |  | 5 |  |  |  |  |  |  |  |  |  | 14 |  |  |  | 12 | 31 |
| **E** | *r* |  |  | 0.883 |  |  |  |  |  |  |  |  |  | 0.394^e^ |  |  |  | 0.781 | 0.763 |
|  | *F*_ST_ |  |  | 0.058 |  |  |  |  |  |  |  |  |  | 0.031 |  |  |  | 0.007 | 0.029 |
|  | *P* value |  |  | 0.0005 |  |  |  |  |  |  |  |  |  | 0.003 |  |  |  | 0.620 | 0.0001 |
| **F** | #alleles | 7 |  |  |  |  |  |  |  |  |  | 11 |  |  |  |  |  |  | 18 |
|  | *r* | 0.988 |  |  |  |  |  |  |  |  |  | 0.887 |  |  |  |  |  |  | 0.958 |
|  | *F*_ST_ | 0.004 |  |  |  |  |  |  |  |  |  | 0.011 |  |  |  |  |  |  | 0.008 |
|  | *P* value | 0.023 |  |  |  |  |  |  |  |  |  | 0.0001 |  |  |  |  |  |  | 0.0001 |

1: *BM3517*, 2: *BM4028*, 3: *ETH010*, 4: *ETH225*, 5: *INRA006*, 6: *INRA128*, 7: *TGLA227*, 8: *TGLA263*, 9: *CSSM019*, 10: *DIK020*, 11: *TGLA057*, 12: *BM0719*, 13: *BM1824*, 14: *BM3205*, 15: *ILSTS026*, 16: *TGLA159*, 17: *SPS115*. Underlined: significance (α = 0.05) of individual locus (after Bonferroni) or μsat set. a: Overall Pearson *r* obtained by correlating frequencies of all alleles across loci, b: low correlation due to high frequency of allele 138 in set C (0.157 vs. 0.047 in set B). Pearson *r* = 0.886 without this allele. c: low correlation due to low frequency of allele 188 in set C (0.072 vs. 0.218 in set B). Pearson *r* = 0.861 without this allele. d: when #alleles ≤ 4: frequencies of the most common allele (> 0.54) differ by a factor < 1.03. e: Pearson *r* = 0.781 with the pooled sample of Mana Pools NP, Nyakasanga, Gorongosa NP and Marromeu GR, which are located 730 km further south. *F*_ST_ values and G-statistics derived *P* values (9999 randomizations) were estimated with the Genalex add-in for Excel (version 6.503).

**Allele size changes relative to microsatellite set A (set A minus other set)**

B: μsat 1: -8; μsat 2, 6: -2, μsat 5: -11; μsat 8: -1; μsat 9: if ≤ 143: -5, if ≥ 146: -4, 144-145: not observed in any population; μsat 10: if ≤ 181: -3, if ≥ 182: -2; μsat 11: if ≤ 97: -6, if ≥ 98: -5; μsat 12: if ≤ 147: -1, if ≥ 150: -2, 148-149: not observed in any population; μsat 14: if ≤ 210: -4, if ≥ 213: -5, 211-212: not observed in any population; μsat 15: if ≤ 149: +2, if ≥ 152: +1, 150-151: not observed in any population; μsat 16: -5.

C: μsat 1, 2, 6: -2; μsat 9: if ≤ 135: -3, if ≥ 139: -5, 137: not observed in any population; μsat 10: if ≤ 195: -3, if ≥ 197: -5. Allele sizes 195 and 197 resulted both in an adjusted allele size of 192. For the allele size range 195-199 it was unclear whether the shift should be -3 or -5, because allele size 197 was only observed in Laikipia NP at low frequency (frequency = 0.029). This inconsistency did not affect our analyses as allele sizes 193-201 (190-196 after adjustment) were rare in KNP (i.e., not considered as an MDTA or wild-type associated allele); μsat 11: if ≤ 87: 0, if ≥ 91: -2; μsat 12: if ≤ 145: -1, if ≥ 147: -3 Allele sizes 145 and 147 resulted both in an allele size of 144. Allele size 145 was only observed in Nairobi NP at low frequency (frequency = 0.026). It was unclear whether allele size 145 should be decreased by -1 or -3. This inconsistency did not affect our results as both allele size 142 and allele size 144 were rare in KNP (i.e., not considered as an MDTA or wild-type associated allele); μsat 14: no change; μsat 15: if ≤ 150: -1, if ≥ 152: -3, Allele sizes 150 and 152 were both adjusted to an allele size of 149, which constitutes a male-deleterious-trait-associated allele. These size shifts represent a conservative choice as they resulted in a more positive Pearson *r* when correlating allele frequencies against latitude (i.e., less significant allele-frequency cline).; μsat 16: -3.

D: μsats 2-4, 6-8, 10, 13, 15: no change; μsat 5: -4; μsat 9: if ≤ 148: no change, if ≥ 152: -2, 150: not observed in any population; μsat 17: if ≤ 233: -4, if ≥ 237: -6, 235: not observed in any population.

E: μsat 3: no change; μsat 13: if 167: +2, if ≥ 175: no change, 169-173: not observed in Serengeti NP; μsat 17: if ≤ 234: -1, if ≥ 236: +1

F: μsat 1: -5, : μsat 11: -2
