# Supplementary material for: A continent-wide high genetic load in African buffalo revealed by clines in the frequency of deleterious alleles, genetic hitchhiking and linkage disequilibrium
Source: PLoS One. 2021 Dec 9;16(12):e0259685. doi: 10.1371/journal.pone.0259685 (PMC8659316; doi:10.1371/journal.pone.0259685)
Supplement: S1 Fig — (DOCX) [file pone.0259685.s010.docx]

**Figure S1**: Increase of pairwise *F*_ST_ with geographic distance

Regressing pairwise multilocus *F*_ST_, estimated with FSTAT 2.9.3 [1], against geographic distance explains 65% of the variation among populations in East and southern Africa (*N*_μsats_ = 5-17, *N*_pop._ = 27; purple and yellow datapoints combined), excluding Nairobi NP and HiP (blue and orange datapoints, respectively) and weighted by ‘square root of number of genotyped individuals per population pair X number of shared genotyped loci per population pair’. The amount of variation explained increases to 78% when selecting the upper quartile with respect to sample weight (i.e., selecting the most accurate measurements; yellow datapoints). Pairs including HiP or Nairobi NP show relatively high *F*_ST_ values. These are probably due to a historical bottleneck in case of HiP, and a small population size and limited connectivity with other populations in case of Nairobi NP (HiP: ≤ 75 individuals between 1895 and 1930, Nairobi NP: current census size ~150 and genetic discontinuities in a Barrier analysis) [2, 3].


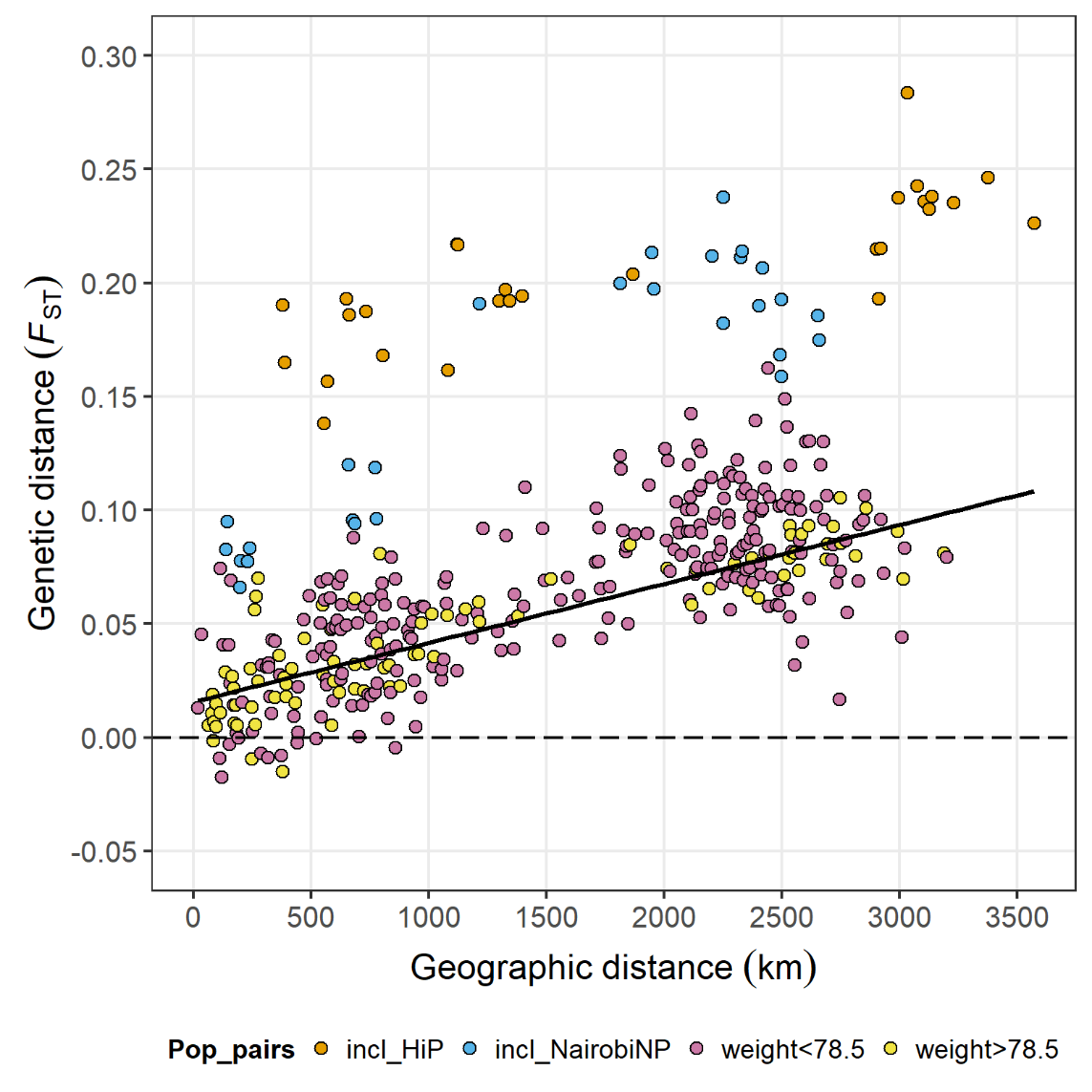


Figure S1: Increase of pairwise *F*_ST_ with geographic distance (isolation by distance)

Yellow datapoints: *R*^2^ = 0.78, purple datapoints: weight: *R*^2^ = 0.54. *F*_ST_ values were based on unpooled samples. Populations with ≤ 3 individuals genotyped, admixed populations or with only two microsatellites analysed were excluded (Benoué NP, Lopé NP, Nyakasanga, Mangua, Caprivi Strip, Save Valley Conservancy).

**References**

1. Goudet J. FSTAT, a program to estimate and test gene diversities and fixation indices (version 2.9.3). Updated from Goudet (1995). 2001.

2. Heller R, Okello JBA, Siegismund H. Can small wildlife conservancies maintain genetically stable populations of large mammals? Evidence for increased genetic drift in geographically restricted populations of Cape buffalo in East Africa. Mol Ecol. 2010;19(7):1324-34. doi: 10.1111/j.1365-294X.2010.04589.x. PubMed PMID: WOS:000275761300008.

3. van Hooft P, Getz WM, Greyling BJ, Bastos ADS. A natural gene drive system influences bovine tuberculosis susceptibility in African buffalo: possible implications for disease management. PLoS ONE. 2019;14(9):e0221168.
