# Supplementary material for: A continent-wide high genetic load in African buffalo revealed by clines in the frequency of deleterious alleles, genetic hitchhiking and linkage disequilibrium
Source: PLoS One. 2021 Dec 9;16(12):e0259685. doi: 10.1371/journal.pone.0259685 (PMC8659316; doi:10.1371/journal.pone.0259685)
Supplement: S2 Fig — (DOCX) [file pone.0259685.s011.docx]

**Figure S2**: Multilocus-*H*_e_ cline based on twelve microsatellites per locality


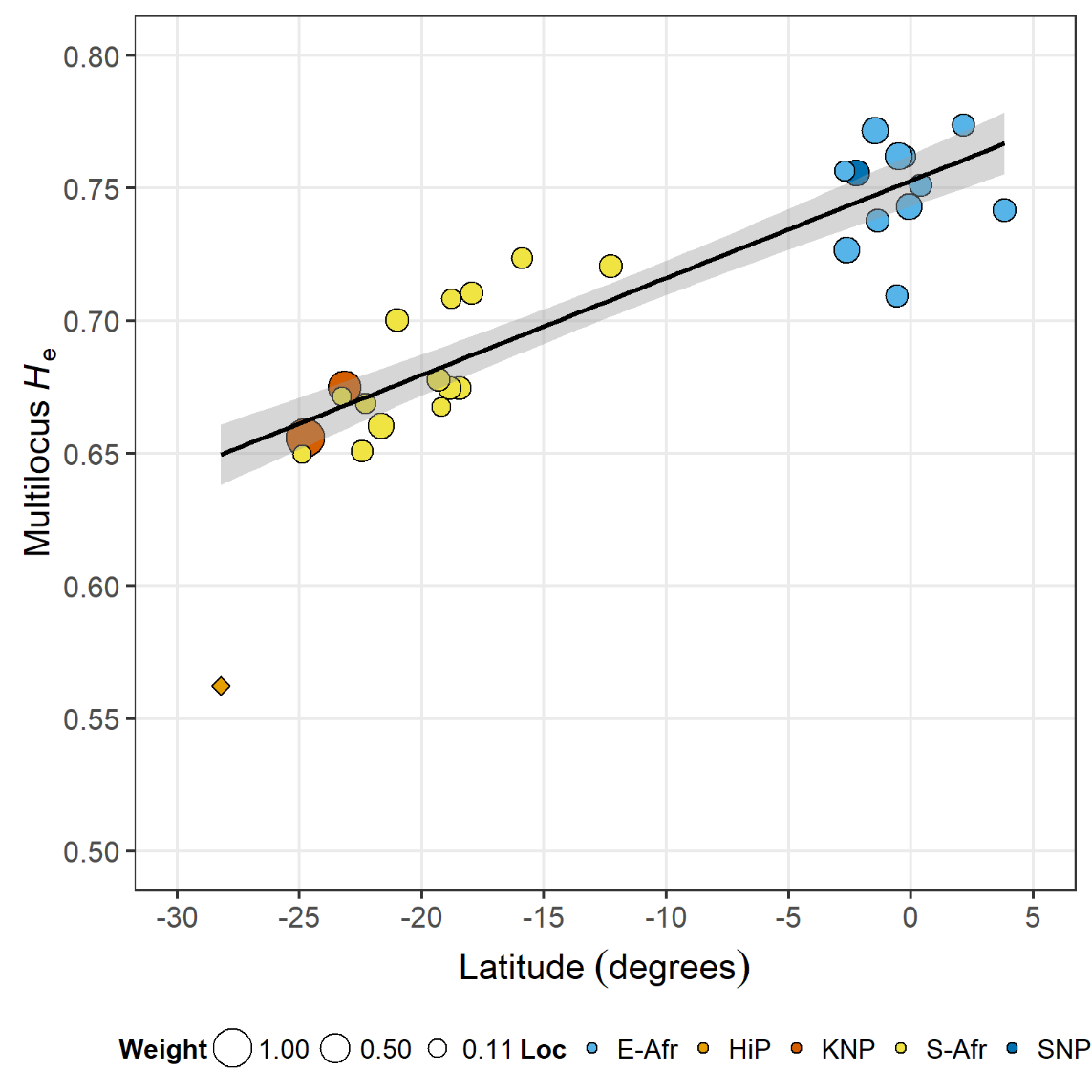


Figure S2: Multilocus-*H*_e_ cline

Scatter plot and regression line with 95% confidence interval, adjusted *R*^2^ = 0.85 (excluding Hluhluwe-iMfolozi Park). Predicted multilocus *H*_e_ decreased by 14% (95% CI: 11%, 16%) at 24.9 °S compared to 3.9 °N. *H*_e_: expected heterozygosity, HiP: Hluhluwe-iMfolozi Park, KNP: Kruger NP, SNP: Serengeti NP. Red, orange, yellow and dark blue data points: multilocus *H*_e_ based on microsatellites *ABS010*, *AGLA293*, *BM1824*, *BM4028*, *ETH010*, *INRA006*, *INRA128*, *CSSM019*, *DIK020*, *ILSTS026*, *SPS115* and *TGLA263*. Light and dark blue data points: multilocus expected *H*_e_ based on microsatellites *ABS010*, *AGLA293*, *BM3517*, *BM4028*, *INRA128*, *BM0719*, *BM3205*, *CSSM019*, *DIK020*, *ILSTS026*, *TGLA057* and *TGLA159*. SNP: analysed with both microsatellite sets. For *ABS010* and *AGLA293* in northern KNP the weighted average of microsatellite sets B and D was used (all other microsatellites set A only). Multilocus *H*_e_ of the northern localities (light blue data points) was multiplied with 0.972, which was the ratio between the two microsatellite sets in Serengeti NP; the only locality in East Africa analysed with both sets.
