# Supplementary material for: A continent-wide high genetic load in African buffalo revealed by clines in the frequency of deleterious alleles, genetic hitchhiking and linkage disequilibrium
Source: PLoS One. 2021 Dec 9;16(12):e0259685. doi: 10.1371/journal.pone.0259685 (PMC8659316; doi:10.1371/journal.pone.0259685)
Supplement: S3 Fig — (DOCX) [file pone.0259685.s012.docx]

**Figure S3**: Multilocus-*H*_e_ cline based on seven microsatellites analysed in both East and southern Africa (microsatellite sets A, B and D) plus *ABS010* and *AGLA293*


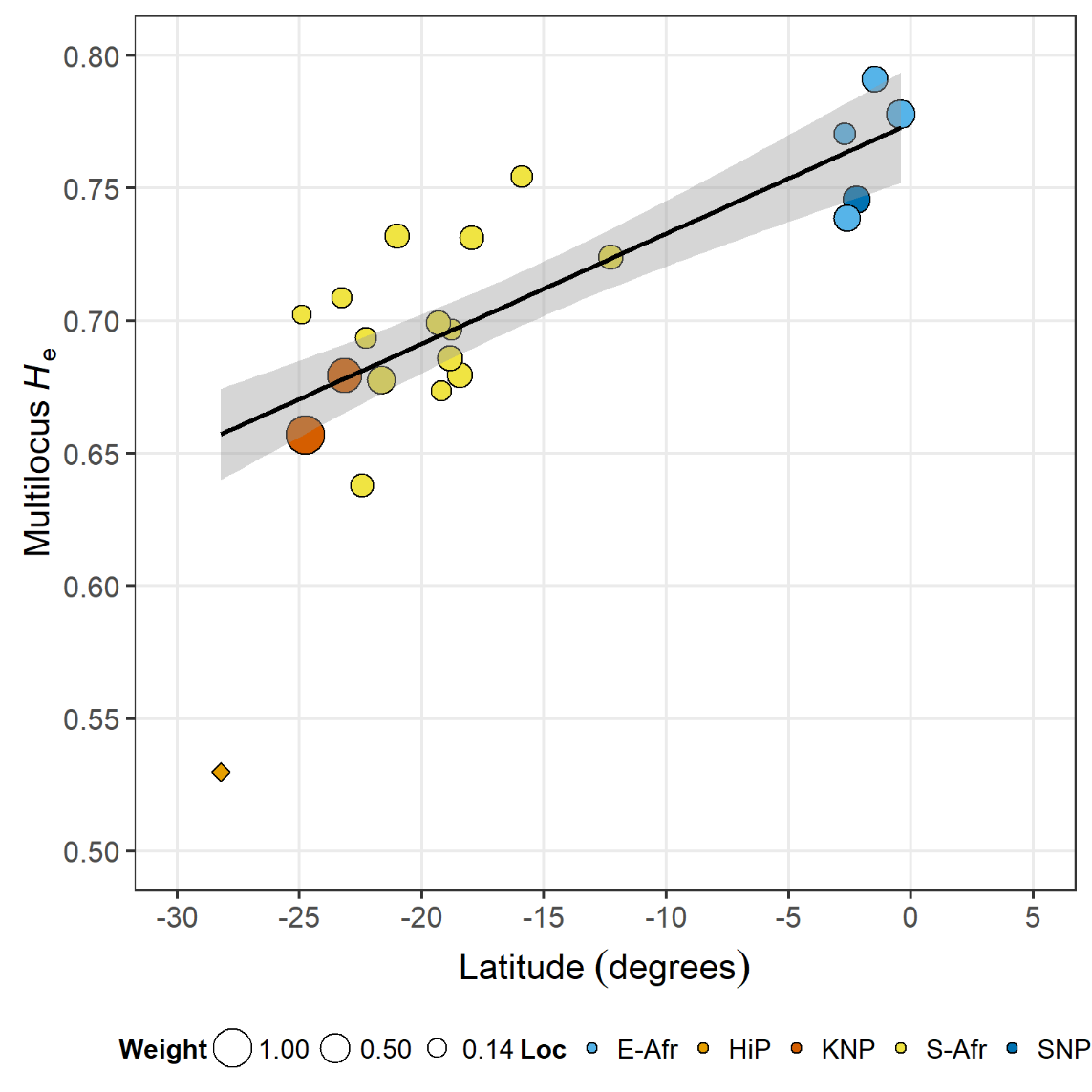


Figure S3: Multilocus-*H*_e_ cline

Scatter plot and regression line with 95% confidence interval, adjusted *R*^2^ = 0.74 (excluding HiP). Predicted multilocus *H*_e_ decreased by 13% (95% CI: 9%, 17%) at 24.9 °S compared to 0.4 °S. *H*_e_: expected heterozygosity, HiP: Hluhluwe-iMfolozi Park, KNP: Kruger NP, SNP: Serengeti NP. Multilocus *H*_e_ based on microsatellites *ABS010*, *AGLA293*, *BM4028*, *INRA006*, *INRA128*, *CSSM019*, *DIK020*, *ILSTS026* and *TGLA263*. For *ABS010* and *AGLA293* in northern KNP the weighted average of microsatellite sets B and D was used (all other microsatellites set A only).
