# Supplementary material for: A continent-wide high genetic load in African buffalo revealed by clines in the frequency of deleterious alleles, genetic hitchhiking and linkage disequilibrium
Source: PLoS One. 2021 Dec 9;16(12):e0259685. doi: 10.1371/journal.pone.0259685 (PMC8659316; doi:10.1371/journal.pone.0259685)
Supplement: S4 Fig — (DOCX) [file pone.0259685.s013.docx]

**Figure S3**: Correlation between per-locus southern/northern KNP *H*_e_ ratio and *H*_e_-latitude Pearson correlation, based on 19 microsatellites


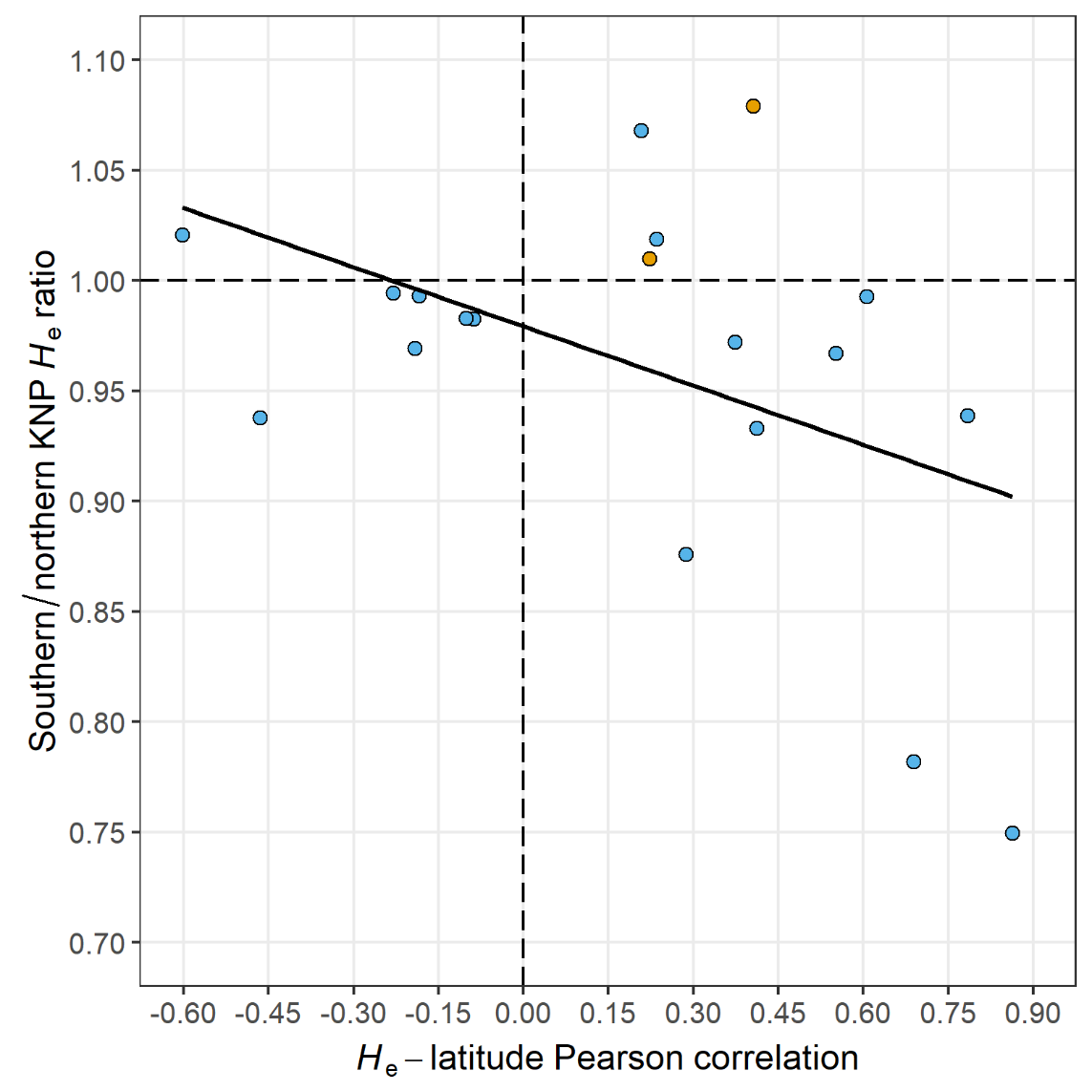


Figure S3: Correlation between per-locus southern/northern KNP *H*_e_ ratio and *H*_e_-latitude Pearson correlation

Spearman *ρ* = -0.51, *P* = 0.025, *N* loci = 19. *H*_e_: expected heterozygosity, KNP: Kruger NP. The correlation is based on microsatellites: *ABS010* (orange data point), *AGLA293* (orange data point), *BM0719*, *BM1824*, *BM3517*, *BM3205*, *BM4028*, *CSSM019*, *DIK020*, *ETH010*, *ETH225*, *ILSTS026*, *INRA006,* *INRA128*, *SPS115*, *TGLA057*, *TGLA0159*, *TGLA227* and *TGLA263*. For *ABS010* and *AGLA293* in northern KNP the weighted average *H*_e_ of microsatellite sets B and D was used (all other microsatellites set A only). The correlation was weighted by the square-root of the total sample size in KNP because of the relatively small average sample sizes in KNP, particularly southern KNP, for *ABS010* and *AGLA293* (northern KNP: 46 vs. 134 for the other microsatellites, southern KNP: 16 vs. 308 for the other microsatellites).
